# Soil Bacterial Community Response to Differences in Agricultural Management along with Seasonal Changes in a Mediterranean Region

**DOI:** 10.1371/journal.pone.0105515

**Published:** 2014-08-21

**Authors:** Annamaria Bevivino, Patrizia Paganin, Giovanni Bacci, Alessandro Florio, Maite Sampedro Pellicer, Maria Cristiana Papaleo, Alessio Mengoni, Luigi Ledda, Renato Fani, Anna Benedetti, Claudia Dalmastri

**Affiliations:** 1 ENEA (Italian National Agency for New Technologies, Energy and Sustainable Economic Development) Casaccia Research Center, Technical Unit for Sustainable Development and Innovation of Agro-Industrial System, Rome, Italy; 2 Consiglio per la Ricerca e la Sperimentazione in Agricoltura - Research Centre for the Soil-Plant System, Rome, Italy; 3 Laboratory of Microbial and Molecular Evolution, Department of Biology, University of Florence, Florence, Italy; 4 Dipartimento di Agraria, University of Sassari, Sassari, Italy; Argonne National Laboratory, United States of America

## Abstract

Land-use change is considered likely to be one of main drivers of biodiversity changes in grassland ecosystems. To gain insight into the impact of land use on the underlying soil bacterial communities, we aimed at determining the effects of agricultural management, along with seasonal variations, on soil bacterial community in a Mediterranean ecosystem where different land-use and plant cover types led to the creation of a soil and vegetation gradient. A set of soils subjected to different anthropogenic impact in a typical Mediterranean landscape, dominated by *Quercus suber* L., was examined in spring and autumn: a natural cork-oak forest, a pasture, a managed meadow, and two vineyards (ploughed and grass covered). Land uses affected the chemical and structural composition of the most stabilised fractions of soil organic matter and reduced soil C stocks and labile organic matter at both sampling season. A significant effect of land uses on bacterial community structure as well as an interaction effect between land uses and season was revealed by the EP index. Cluster analysis of culture-dependent DGGE patterns showed a different seasonal distribution of soil bacterial populations with subgroups associated to different land uses, in agreement with culture-independent T-RFLP results. Soils subjected to low human inputs (cork-oak forest and pasture) showed a more stable bacterial community than those with high human input (vineyards and managed meadow). Phylogenetic analysis revealed the predominance of *Proteobacteria*, *Actinobacteria*, *Bacteroidetes*, and *Firmicutes* phyla with differences in class composition across the site, suggesting that the microbial composition changes in response to land uses. Taken altogether, our data suggest that soil bacterial communities were seasonally distinct and exhibited compositional shifts that tracked with changes in land use and soil management. These findings may contribute to future searches for bacterial bio-indicators of soil health and sustainable productivity.

## Introduction

Soil microorganisms play an important role as regulators of major biogeochemical cycles and can significantly affect the ecosystem functioning [1], being involved in organic matter dynamics, nutrient cycling and decomposition processes [2]. The anthropogenic activities affect the diversity of natural habitats modifying the number of species occurring in the environment at the landscape scale. Soil management strongly influences soil biodiversity in agricultural ecosystems. Different practices can alter the below-ground ecosystem, often leading to depletion of soil carbon and loss of biodiversity, and thus affecting the structure of the resident microbial communities [3]. Therefore, characterizing genetic and functional diversity of soil bacterial communities in response to agricultural practices and/or climate is fundamental to better understand and manage the ecosystem processes.

The Mediterranean area is one of the most important biodiversity hotspots in the world and is increasingly threatened by intensive land use [4]. The high environmental diversity that characterizes the Mediterranean region is related to the integration of natural ecosystems and traditional human activities such as the agroforestry practices [5]. The collapse of the traditional agro-silvo-pastoral system that occurred during the past century has led to major changes in the extension of woodlands dominated by typical Mediterranean species, i.e. cork oak (*Quercus suber*) and/or holm oak (*Quercus ilex*) woodlands [6], [7]. Research on the influence of management practices on the biodiversity of these agro-silvo-pastoral systems is increasing but it has focused mostly on plants [8] and vertebrates [5], [9]. In the frame of the Italian Project SOILSINK (Climatic changes and agricultural and forest systems: impact on C reservoirs and on soil microbial diversity), a hilly basin in Gallura (Berchidda site, Sardinia, Italy) was selected as a reference Mediterranean site for studying the influence of land-use changes on diversity, function and seasonal variations of soil microbial communities [10], [11]. The site is within an area of about 1,450 ha and is characterised by extensive agro-silvo-pastoral systems, typical of north-eastern Sardinia (Italy) and similar areas of the Mediterranean basin [12]. The chosen site represents a sustainable balance between human activities and natural resources that have created a landscape of high heterogeneity and cultural value, whose importance has been recognized at the European level [13], [14]. Indeed, it is considered climatically (Mediterranean zone) and pedologically homogeneous with vegetation patterns similar to those called *dehesas* or *montados* of the south-western Iberian Peninsula [15], [16], [17]. In the past, this area was covered by cork-oak forests, which gradually were subjected to increasing under-storey grazing and usage for the extraction of cork. Today, there are different land-use and plant cover types that lead to a soil and vegetation gradient with an ecological progression: from a cork-oak forest undergoing minimum disturbance to managed vineyards with an intensive agricultural practice (grass covered and ploughed), passing through areas with temporary grassland, and pasture.

The different land uses altered soil potential, making possible to discriminate the role of human management on soil functioning. When forests are converted to grasslands, and grasslands turned into agricultural lands, a sharp switch from one type of soil microbial community to another one occurs. Since the ability of an ecosystem to withstand serious disturbances may partly depend on its microbial component(s), characterizing bacterial community composition and/or structure might help to better understand and manipulate ecosystem processes. The aim of the present study was to investigate the effects of soil characteristics and different agricultural managements on soil bacterial community in two seasons. Sampling was carried out in spring and autumn 2007, when the plant cover-growing season usually starts and ends. A combination of culture-based and molecular techniques along with statistical analysis of data obtained was applied to interrogate the diversity, function, and ecology of soil bacterial communities. The results obtained in the present study, along with the other ones obtained within the SOILSINK Project [11], [18], [19], [20], provide useful data on the impact of soil type, cover vegetation, and human activities on the distribution of the bacterial genetic resources in soil communities for this Mediterranean region. Our results confirm that the environments with low inputs (cork-oak forest and pasture) show a more stable soil microbial community than those subjected to increasing human input (vineyards and managed meadow) and suggest that soil bacterial communities are seasonally distinct with compositional shifts that track with changes in land use and soil management.

## Materials and Methods

### Ethics Statement

We carried out the study on the hilly basin in Gallura (Olbia-Tempio municipalities, Sardinia, Italy). Five soils uses were identified in private farms within Berchidda site (40°49′ 15″N, 9°17′ 32″ E) and were obtained. The soil sampling was carried out in the frame of a national research project (SOILSINK Project) and soils used in this study were collected under consent of the landowners. The responsible of the study site was Prof. P.P. Roggero (University of Sassari, Italy). We confirm that our study did not harm the environment and did not involve endangered or protected species. Specific geographic coordinates (referred to World Geodetic System, 1984) of our study area are reported in Table S1.

### Sampling site

The study area (Olbia-Tempio) is representative of the climate, vegetation type and management of some of the most common agro-forestry systems in the Mediterranean basin [16].

The Berchidda site is made up of hydromorphic and granitic soil with a loamy sand texture. The altitude ranges from 275 m to 300 msl. This area is referred to as a meso-thermo Mediterranean, subhumid phytoclimatic belt with a mean annual rainfall of 862 mm and the mean annual temperature of 13.8°C [10]. In the past, the Berchidda area was covered by cork-oak forests (dominated by *Quercus Suber* L.), which were subjected to intense usage for the extraction of cork. Today, there are five main different land-use units close together: cork-oak forest (CO), hayland-pasture rotation (PA), managed meadow (MM), and tilled (TV) and grass covered vineyard (CV). The cork-oak formation, pasture, and managed meadow have been converted to the current use and maintained unchanged for more than 30 years, whereas the non-tilled cover cropped vineyard and the tilled one were planted in 1985 and 1994, respectively. The five soils are located inside an area of 161.5 km^2^. Detailed characteristic and management of the five soils have been previously described [12], [20], [21], [22], [23].

### Pedological characterization of the study area

The pedogenic substrate of the study area consists of medium-grained granite, affected by localized presence of veins of quartz and porphyry. The morphology of the Berchidda area varies from flat to undulating. The processes of soil erosion by water channeled are evident only in the short-term forage crops made on soil with more than 15% of slope. All profiles have a horizons sequence of the type A-Bw-C or A-Bw-BC-C or more rarely, A-Bw-C-R (Table S1). The power of the profiles, limited to horizons A and Bw, varies from a minimum of 38 cm to a maximum of 100 cm. In three soil profiles in TV, the sequence between the horizon A and Bw and the substrate is gradually altered by the presence of a horizon BC, characterised by coarse texture, the power of which varies from about 30 cm to 90 cm. Direct contact with the unaltered rock, R horizon, was observed only in one profile in CV. The prevailing textural classes are sandy and sandy loam. The content of organic substance in the horizons A is never very high, with average value around to about 3%. The maximum values of 11.8% and 8.6% were observed on the A horizons in two soil profiles in CO. The exchange complex, in agreement with the reduced content in clays, is never high. The degree of base saturation is predominantly less than 60% (Dystric conditions of the USDA Soil Taxonomy). The profiles with the exchange complex with a degree of base saturation below 60% in all horizons between 25 and 75 cm (TV, CV, MM, and PA) were classified as Typic Dystroxerepts [24]. The other one (CO) was classified as Lithic Xerorthents.

### Soil sampling

**Figure 1:**
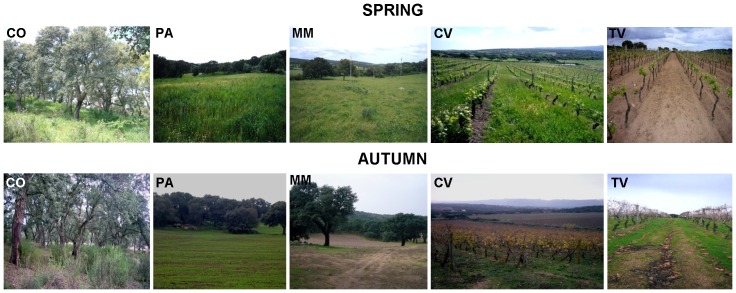
Long-term effects of different land-use with increasing level of intensification in spring and autumn. Both pasture and managed meadow included spotted cork oak trees, which are key components of the Dehesatype landscape typical of this area of Sardinia. The cork-oak formation, pasture, and managed meadow have been converted to the current use and maintained unchanged for more than 30 years, whereas the non-tilled cover cropped vineyard and the tilled one were planted in 1985 and 1994, respectively. From the left to right: cork-oak forest (CO), hayland pasture rotation (PA), managed meadow (MM), grass covered vineyard (CV), tilled vineyard (TV).

Five soil replicates were collected from bulk soil of the five different managements (CO, PA, MM, CV, and TV) (Fig. 1). Soils were collected in May and November 2007. After removal of litter layer, soil core samples (50 to 100 g; diameter, 5 cm) were taken from each of the five locations, using a 5-on-dice sampling pattern with ca. 70 m distance between each sampling point. Sampling was performed at 20 cm depth, where most microbial activity is known to occur [25], [26]. In the vineyard soils, samples from along the rows and between the rows were pooled together to form a field replicate. The other soil samples (CO, PA, MM) were collected out of trees influence. At each season and for each soil type, five randomly field replicates were collected for a total of 25 soil samples (5 replicates × 5 land uses), each one being a composite sample of five soil cores. A total of 50 composite samples were taken for the two seasons. Soil samples were immediately sieved (<2 mm) to remove fine roots and large organic debris, air dried, and transported to the labs for microbiological analysis. The moisture content was adjusted to 60% of their water holding capacity (WHC) and soil samples were then left to equilibrate at room temperature in the dark for one day prior to analyses, in order to restore, within limits [27], the microbial activity of air- dried soils to that of soils in the field.

### Chemical and biochemical analyses of soil samples

The chemical and biochemical analyses were performed on three replicates for each land use for a total of 15 soil samples (3 replicates × 5 land uses), each one being a composite sample of five soil cores. Total organic carbon (C_org_) was estimated after oxidation with K_2_Cr_2_O_7_ and subsequent titration of unreduced Cr_2_O_7_^2−^ with Fe(NH_4_)_2_(SO_4_)_2_, as reported by Springer and Klee [28]. Soil Organic Matter (SOM) was determined by C_org_ multiplied by 1.724 van Belem coefficient. The C_org_ fractionation was set up as reported by Ciavatta and co-workers [29]. In particular, solid samples were extracted at 65°C for 24 h using 0.1 mol l^−1^ NaOH plus 0.1 mol l^−1^ Na_4_P_2_O_7_ solution (1∶50, solid:liquid ratio). The samples were then centrifuged at 5000×*g* and the supernatants were filtered through a 0.20-µm Millipore filter (Millipore, Billerica, MA) (total extractable C, C_ext_). The humic-like acid (HA) fraction was separated from the fulvic-like acid (FA) and the non-humified carbon fractions (NHC) by precipitation after acidification of the alkaline solution (supernatant) to pH<2. Chromatography on a column of polyvinylpyrrolidone (PVP, Aldrich, Germany) was used to separate the NHC from the FA. The FA was then combined with the HA to obtain total humified fraction (HA+FA). Total extractable C (C_ext_ %), humic and fluvic acid C (C_HA+FA_ %) were determined by the dichromate oxidation method. The non-humified carbon (C_NH_) was determined as the difference between C_ext_ and C_HA+FA_. Humification indexes HI, DH, and HR were determined according to previous works [29], [30].

Microbial biomass C (C_mic_) was determined by the fumigation-extraction method of Vance and co-workers [31] with some slight modifications. The measurements were performed on air-dried soils, pre-conditioned by a 10-d incubation in open glass jars, at –33 kPa water tension, and 30°C. The incubation was employed for restoring, within limits [27], the microbial activity of air-dried soils to that of soils in the field. Four replicates of each soil sample were used. Average values are given in mg C kg^–1^ of soil. For measuring microbial respiration, 20 g (oven-dry basis) of moist sample were placed in 1 L stoppered glass jars. The CO_2_ evolved was trapped, after 1, 2, 4, 7, 10, 14, 21, 28 days of incubation, in 2 ml 1 M NaOH and determined by titration of the excess NaOH with 0.1 M HCl [32].

Non-linear least square regression analysis was used to calculate parameters affecting C mineralization from daily CO_2_ evolution data (Stat Win 4.0 for Windows). The best fit was obtained with the exponential model of CO_2_-C accumulation according to the negative exponential decay model:
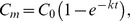
where C_m_ is the cumulative value of mineralized C during *t* days, *k* is the rate constant, and C_o_ is the potentially mineralizable C [33]. The CO_2_ emitted in 28 days of incubation was used as cumulative respiration (C_cum_). The CO_2_ evolved during the 28th day of incubation was used as the basal respiration value (C_bas_). Microbial indices were calculated as follows [34], [35], [36]:



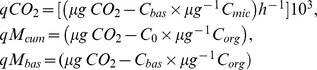



### Recovery of cultured bacterial cells from soil samples

Soil bacterial community analysis was performed on five soil replicates for each land use, as described above. Bacterial cell extraction was performed according to the recommendations of Smalla et al. [37] with minor modifications. Briefly, 1 g of soil was placed in a sterile 15 ml plastic tube containing 10 ml of phosphate buffered saline (PBS, pH 7.0). This mixture was homogenized for 30″ at low speed by using the Ultra-Turrax Thyristor Regle 50 (Janke & Kunkel IKA-Labortechnik). After homogenization, suspension was placed into a Erlenmeyer flask (100 ml) containing 10 g of glass beads (0,2 mm) and shaken for 1 h at 180 r.p.m. and 28°C to disperse bacteria. The flasks and glass beads were autoclaved for 20 min at 121°C before use. The soil suspension was transferred into a sterile 15 ml plastic tube and serially diluted with sterile saline solution (9 g l^−1^NaCl) from 10^−1^ up to 10^−7^. The, 100 µl aliquots of serially diluted soil suspensions were plated in triplicate on 0.1 Triptic Soy Broth (TSB; Difco) amended with 15 g l^−1^ agar (0.1 TSA) and 100 g µl^−1^ of cycloheximide (Sigma) to inhibit fungal growth. Plates were incubated at 28°C for 6 days.

### Growth strategy and total bacterial populations

To determine the changes in the structure of culturable fraction of soil bacteria, the r/K-strategy concept proposed by De Leij and co-workers [38] was used. Bacterial colonies appearing within 48 h were designated as r-strategists, and the remaining as K-strategists. Colonies were enumerated at 1, 2 and 6 days of growth on 0.1 TSA; in this way, three counts (or classes) were generated per sample. Plates containing between 30 and 300 colonies were then selected for enumeration. Total bacterial counts obtained were expressed as colony forming units (CFU) per gram of soil. Distribution of bacteria in each class as a percentage of the total counts gave insight into the distribution of r- and K-strategists in each sample.

To evaluate the changes in the biodiversity of bacterial populations in soils, the eco-physiological (EP) index [38] was used. The EP index of each soils tested was calculated using the equation:
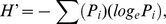



where *P_i_* represents the CFU on each day (1, 2 and 6 days of incubation) as a proportion of the total CFU in that sample after 6 days incubation, i.e., the proportion of colonies appearing on counting day *i* (*i* = 1, 2, 6) with EP_min_  = 0. Higher values of EP index imply a more even distribution of proportions of bacteria developing on different days (i.e., different classes of bacteria).

### Terminal-Restriction Fragment Length Polymorphism (T-RFLP)

DNA was extracted from soil samples by using the FastDNA SPIN Kit for Soil (QBiogene). Terminal-Restriction Fragment Length Polymorphism (T-RFLP) was performed on 16SrRNA genes amplified from extracted DNA with primer pairs P0 and P6 as previously reported [39], [40]. Purified amplification products were digested separately with restriction enzymes *Rsa*I and *Msp*I and digestions were resolved by capillary electrophoresis on an ABI310 Genetic Analyzer (Applied Biosystems, Foster City, CA, USA) using LIZ 500 (Applied Biosystems) as size standard. T-RFLP analysis was performed as previously reported [41]. Diversity indices were calculated with PAST software [42] as previously reported [19], taking into account peak intensities of T-RFLP fragments. Taxonomic interpretation of T-RFLP profiles was performed by querying the Ribosomal Database Project Database by using MiCA3 web tool (http://mica.ibest.uidaho.edu/), as previously described [40].

### Culture-dependent DGGE (CD DGGE) analysis

Culture-dependent DGGE (CD DGGE) fingerprinting of 16S rRNA gene was used to characterize mixed bacterial communities recovered on agar plates.

*Collection of cultured bacterial communities for DGGE analysis*. Cultured bacterial communities were collected following procedure proposed by Duineveld and co-workers [43] with minor modifications. Briefly, after one week of incubation at 28°C, colonies were removed from plates containing between 100 and 1000 colonies by adding 3.0 ml sterile physiological solution (0.9% NaCl) on each plate and scraping off all grown colonies with a sterile Drigalski spatula. The cell suspensions thus obtained were aliquoted into 1.5 ml Eppendorf tubes and centrifuged at 8,000 r.p.m. for 10 minutes. The pellets were stored at −80°C for subsequent DNA extraction and PCR-DGGE analysis.

*DNA extraction and PCR amplification of 16S rRNA genes.* Genomic DNA was extracted with sodium dodecyl sulfate-proteinase K lysis buffer, followed by a treatment with cetyltrimethylammonium bromide (CTAB) as described in *Current Protocols for Molecular Biology* [44]. Briefly, the pellet was resuspended in 567 µl of TE [10 mM Tris-HCl - 1 mM EDTA (pH 8.0)] buffer, and glass beads 0.3 mm in diameter (250 mg) were added, followed by bead beating for 20 s. Then, 30 µl of 10% sodium dodecyl sulfate and 3 µl proteinase K 20 mg/ml (Sigma) were added and samples were incubated at 37°C for 1 h. Glass beads were removed by centrifugation 2 min at 2,800 rpm and samples were then incubated at 65°C for 10′ with100 µl of 5 M NaCl prepared with sterile water and 80 µl of CTAB/NaCl (10% CTAB in 0.7 M NaCl). Following incubation, extracts were purified by using phenol/phenol-chloroform/isoamyl alcohol (49.5∶49.5∶1) extraction and DNA was recovered by isopropanol precipitation at 4°C o/n. Pelleted DNA was washed twice with cold 70% ethanol, allowed to air dry, and re-suspended in 50 µl of sterile water. Quantity and purity of DNA were checked by NanoDrop (NanoDrop Technologies, USA) and gel electrophoresis. The DNA samples were stored at –20°C until required for use.

The 16S rRNA gene was amplified using 20 ng of lysate suspension and the universal bacterial primers P0 and P6 [45]. Dilutions 1∶100 (2 µl) of the1450 bp PCR products were then used as template for the second PCR amplification with the forward primer 63F (5′- AGGCCTAACACATGCAAGTC -3′), with a GC clamp (5′-CGCCCGCCGCGCGCGGCGGGCGGGGCGGGGGCACGGGGGG -3′) incorporated at the 5′ end, and the reverse primer 518R (5′-ATTACCGCGGCTGCTGG-3′), to produce 495 bp fragments suitable for DGGE analysis [46]. Both PCR reactions were performed in Qiagen Taq buffer (10X) containing 1.5 mM MgCl_2_, with 150 ng of each primer, 250 µM (each) deoxynucleoside triphosphates, and 0.5 U of *Taq* DNA polymerase (Qiagen, Hilden, Germany) in a 25 µl reaction volume. Cycle parameters for PCR with the primer pairs P0–P6 and 63F-GC and 518R were previously described by Di Cello and co-workers [45] and El Fantroussi and co-workers [46], respectively.

*Denaturing Gradient Gel Electrophoresis*. 16S rRNA gene amplicons were separated by double gradient denaturing gradient gel electrophoresis (DG-DGGE) as described by Cremonesi et al. [47], in a DCode universal mutation detection system (Bio-Rad, CA, USA). Separation of purified PCR products (700 ng) was achieved in6%–12% polyacrylamide (acrylamide: N,N-methylenebisacrylamide, 37.5∶1) gels containing an increasing linear gradient of denaturants ranging from 30% to 60% (100% denaturant corresponds to 7 M urea and40% deionized formamide). Each gel also included marker lanes represented by DGGE profiles containing a large number of discrete bands spanning the entire gradient, suitable for within- and between-gel alignment. Electrophoresis were carried out for 16 h at 75 mV in 1X TAE buffer at 60°C, stained with 50 µg/ml ethidium bromide for 30 min, destained in water and photographed with the UVIpro Platinum Gel Documentation System (GAS7500/7510; Eppendorf, Cambridge,UK).

### Cluster analysis and diversity indices

Quantity One software package (Bio-Rad) and Phoretix 1D PRO software (Phoretix International, Newcastle upon Tyne, United Kingdom) were used for CD-DGGE profile analysis. The cluster analysis and dendrogram generation were carried out by using the Phoretix 1D Pro software according to the manufacturer’s instructions (Phoretix International, Newcastle upon Tyne, United Kingdom). Bands of CD-DGGE patterns were aligned and normalized using reference lanes. Background noise was subtracted by rolling ball algorithm with a radius of 50 pixels; the automatic band detection was performed with a minimum slope of 200 and a noise reduction of 10, and peaks smaller than 2% of the maximum peak were discarded. Bands were manually corrected and matched to create an absent/present binary matrix. The similarity between the band patterns was calculated using the Dice coefficient and the clustering analysis was performed with the unweighted pair group method with arithmetic averages (UPGMA) to generate a dendrogram by using mathematic averages algorithm programs integral to the Phoretix 1D Pro software. Coefficient of cophenetic correlation was used to measure the consistency of clusters.

DGGE banding data were used to estimate three diversity indices by treating each band as an individual operational taxonomic unit (OTU). The number of DGGE bands present in each sample was used to measure the Richness index (*R*). The Shannon-Weaver index of general diversity (*H*′) [48] and the Simpson index of dominance (*D*) [49] were calculated from the number of bands present and the relative intensities of each band (*P_i_*) in each lane. Relative signal intensities of detected bands, in each gel track, were determined by using the Quantity One software package (Bio-Rad) and calculated from the peak area of the densitometric curves.

The Shannon-Weaver diversity (*H’*) was calculated using the following equation:
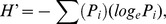



where *P_i_* (the proportion of abundances of the i^th^ band) is measured as:
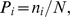
where *n_i_* is the peak height of a band, and *N* is the sum of all peak heights in the densitometry profiles.

The Simpson index (D) was calculated with the formula:
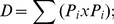
it measures the strength of dominance because it weights towards the abundance of the OTUs and varies inversely with species diversity [50].

### Bacterial isolation and identification

A total of 100 bacterial colonies were randomly picked up for each soil sample from 0.1 TSA plates (containing approximately 50 to 500 colonies), previously used for the determination of the CFU counts and EPI-index, and repeatedly streaked onto 0.1 TSA fresh plates to obtain pure cultures. Isolated colonies were then grown overnight (o/n) in TSB medium at 28°C and 200 r.p.m., and stored at −80°C in 30% glycerol until further analysis. From all five soil samples, 500 colonies were isolated in each season, for a total of 1000 bacterial colonies. A total of 203 colonies with different morphologies (about 20 colonies per each sample) were taken up to investigate their taxonomic affiliation.

Genomic DNA and PCR amplification of the 16S rRNA gene were performed as described above. Sequencing reactions were prepared from PCR products using an Applied Biosystem Big Dye Terminator sequencing kit version 3.1, according to the manufacturer’s instructions and analysed using a 3730 DNA Analyzer Applied Biosystem apparatus. The sequences were compared with those in the GenBank databases by using the BLAST program and Seqmatch tool of the RDP (http://www.ncbi.nlm.nih.gov/BLAST/and http://rdp.cme.msu.edu/, respectively) and aligned with the closest relatives with the Clustal W function of the BioEdit package [51]. Bacterial identification by 16S rRNA gene sequences assignment was performed using the RDP Classification Algorithm (http://rdp.cme.msu.edu/classifier/classifier.jsp).

### Statistical analysis

Bacterial population data (CFU/g of soil) were log transformed and subsequently analysed by one-way ANOVA (STATISTICA, Release 3.0b, Copyright StatSoft Inc., CA, USA). Percentage data of EP index value were *logit*-transformed, as follows:
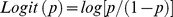
for the proportion *p*, and compared using one-way ANOVA (STATISTICA, Release3.0b, Copyright StatSoft Inc., CA, USA).

Analyses of variance (ANOVA) on biodiversity indexes (Shannon-Weaver, Richness and Simpson), principal component analysis (PCA) and clustering analysis on biochemical data were performed using R packages “stats” and “vegan” (http://cran.r-project.org/and http://cran.r-project.org/web/packages/vegan/index.html). All data clustering were performed using the “UPGMA” algorithm implemented in the “hclust” function of the R “stats” package. Distances among samples were calculated using “Bray-Curtis” distance implemented in “vegan” package as:
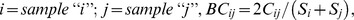
where C_ij_ = sum of the smaller value for species in common between samples i and j, and S_i_ and S_j_ = total number of species in samples i and j, respectively [52]. Variation of biodiversity indexes of cultured bacteria was inspected using ANOVA analysis. Biodiversity indexes were first divided into groups depending on sampling season and different managements of soils and then the analysis was performed. Clustering analysis (UPGMA) on biochemical parameters was performed. Each parameter was first divided in groups, in the same way of previous ANOVA analysis, and averaged. Then, each result obtained was normalized using the maximum-minimum normalization technique in order to make the data comparable. PCA analysis using each biochemical data was performed.

## Results and Discussion

### Effect of land use on soil chemical and biochemical properties

Soil organic matter (SOM) represents a dynamic system influenced by several factors, including climate, clay content, mineralogy and soil management, which all affect the processes of organic matter transformation and evolution in soil [53], [54]. Both soil fertility and stability are related to the organic matter content of soil. Many functions of SOM are due to its more stabilised fractions, humified materials and balance between the labile and the stabilised fractions [55]. Changes in SOM content are related to changes in microbial biomass turnover, because they reflect the balance between rates of microbial organic matter accumulation and rates of organic matter degradation. The extent of organic matter’s organization not only impacts the amount of carbon mineralized but also the type of carbon that is consumed by the microorganisms.

In this study two categories of soil quality indicators were used: organic matter quality indicators and microbial biomass activity indicators (Tables S2 and S3). Our results (Fig. S1) revealed that both land use and sampling season affect the chemical and structural composition of the most stabilised fractions of SOM. A higher content of C_org_ occurred in CO soil in May, and C_ext_ and C_HA+FA_ showed the same pattern, whereas the average values of these parameters were slightly higher in PA soil in November. These results were reflected in the humification parameters, where the humification rate (HR%) can provide quantitative information about the humic substances content normalised with respect to total SOM, while the degree of humification (DH%) provides the amount of the humified carbon in the extracted organic fraction and the humification index (HI) can be considered as an index of soil humification activity as well as of availability of non humified labile fractions [29]. Overall, land use change reduced soil C stocks and labile organic matter at both sampling times except for PA in November. Pasture has a great potential soil organic C stock and, in the long term, grass management systems have nearly equivalent potential to store soil organic C as forest [56]. Potentially mineralizable C (C_0_), which indicates the amount of C in the labile fraction of soil organic matter, decreased over sampling time in all soils, and C_mic_ similarly declined. Cultivated soils are characterised by low microbial activity, mainly due to the disappearance of easily decomposable organic compounds through tillage and soil disturbance. As previously found for chemical properties, the metabolic activity responds to the different land uses; in both May and November samplings, qCO_2_ was higher in CO soil when compared to the others, resulting in increases stress. Hence, unfavourable conditions result in a decrease in the size of the microbial biomass and the efficiency of C substrates degradation, conducting to an increase in respiration rate per unit of microbial biomass [57].

**Figure 2:**
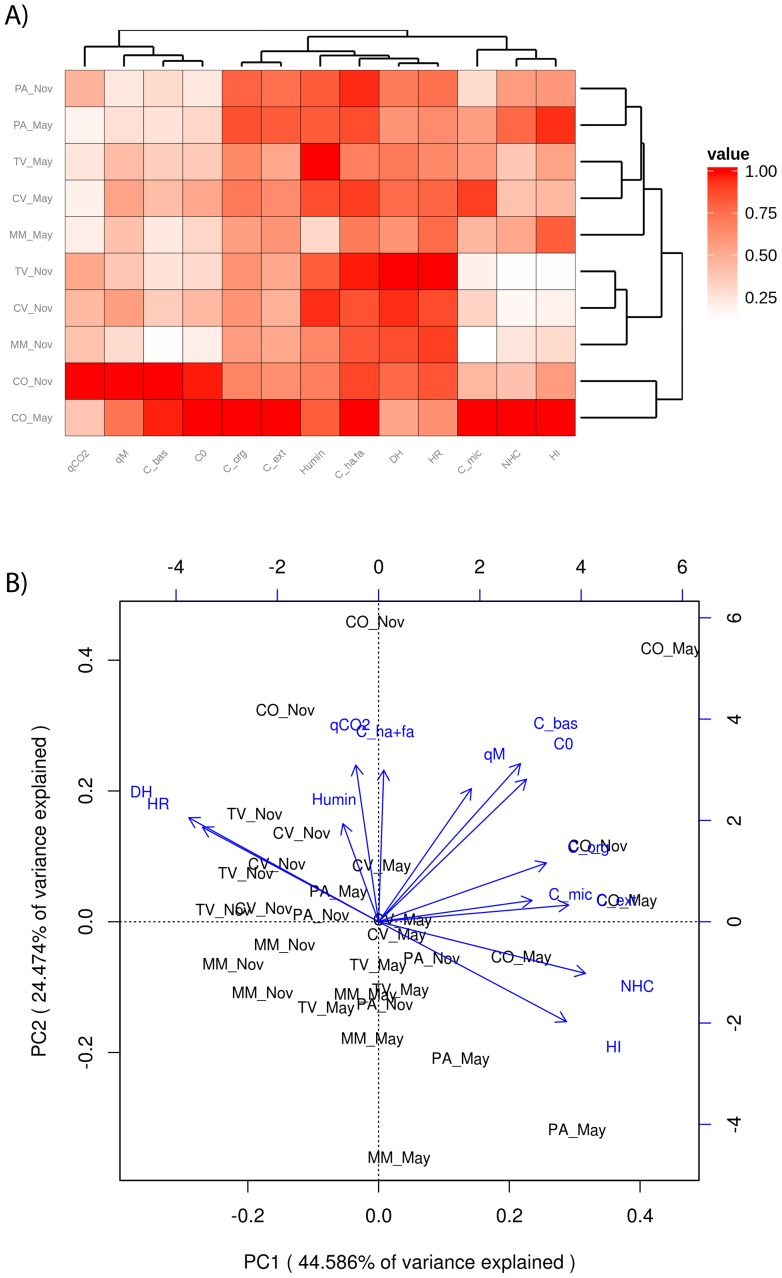
Effect of land-use and season on soil physical-chemical and biological parameters. A) Heat map with hierarchal clustering of physical-chemical and biological parameters across the five Sardinia soils with different land uses at the two different sampling time points (May and November). The heat map was constructed using a maximum-minimum normalization of the data in order to represent each value in a range between 0 and 1. Higher values are represented by darker colors whereas lower ones are represented by lighter colors. CO = cork-oak forest; PA = hayland-pasture rotation; MM = managed meadow; TV =  tilled vineyard; CV =  grass covered vineyard. B) PCA ordination of data (axes 1 and 2) generated from physical-chemical and biological properties of the different types of land use in May and November.

As beneficial and negative effects of soil management practices are strongly linked to microbial activities and regulate soil quality and functioning [58] the relationship existing between soil management practices and variation in chemical and biochemical parameters was evaluated, by performing a clustering analysis (UPGMA) as reported in Tables S2 and S3. The dendrogram obtained was linked to a heat-map representing all the biochemical parameters variation (Fig. 2A). Data analysis revealed that chemical and biochemical parameters clustered in two different groups each of which corresponding to one of the two parameters analyzed (chemical and biochemical). In both sampling times, TV and CV clustered together, suggesting that seasonal change rather than management regime was a major driving force contributing to vineyard soil fertility. Furthermore, according to the UPGMA clustering, CO formed a separate cluster from that of the other soil uses (Fig. 2A). Interestingly, the chemical and biochemical parameters clustered together in both CO and PA, regardless of the season, in contrast to MM, CV and TV, where they clustered separately in relation to the sampling season. This finding suggests that soils subjected to low human inputs (pasture and cork-oak forest) showed a more stable chemical and biochemical soil composition than those with high human input (managed meadow and vineyard).

The different positions of the variables in the plane of the first two principal components, as revealed by PCA analysis (Fig. 2B), indicated that chemical and biochemical parameters were differentially affected by the various land use types. The first and the second principal components (PC1 and PC2) accounted for 44.59% and 24.47% of the total variance in the data, respectively (Fig. 2B). Both chemical and biochemical parameters were positively affected by PC1 except for HR and DH, whereas PC2 was able to discriminate between the two variable sets, with the most of chemical and biochemical parameters being positively and negatively affected, respectively. The above analyses revealed an important effect of land uses on both chemical and biochemical; on the contrary, only biochemical parameters were affected by seasons.

### Influence of land use on soil bacterial community

#### Community structure analysis by EP-index and r-K strategy

Environmental conditions select organisms that either grow rapidly in uncrowded, nutrient-rich conditions (r-strategists), or can efficiently exploit resources in crowded conditions (K-strategists). In this work, we applied the method developed by De Leij et al. [38], who used the r/K-strategy concept for the characterization of soil bacterial communities. First, we compared the microbial community structures found in the different land uses during each of the two seasons. Since sampling was performed at 20 cm depth, where most microbial activity is known to occur [26], the main changes are expected through conversion from one soil management system to another one [59]. Microbial community structure found in the different land uses during each of the two seasons was investigated by means of EP index that is a measure of both richness (i.e. total number of species in the community) and evenness (i.e. how evenly individuals in the community are distributed over the different species) of groups of microorganisms with similar developmental characteristics.

**Figure 3:**
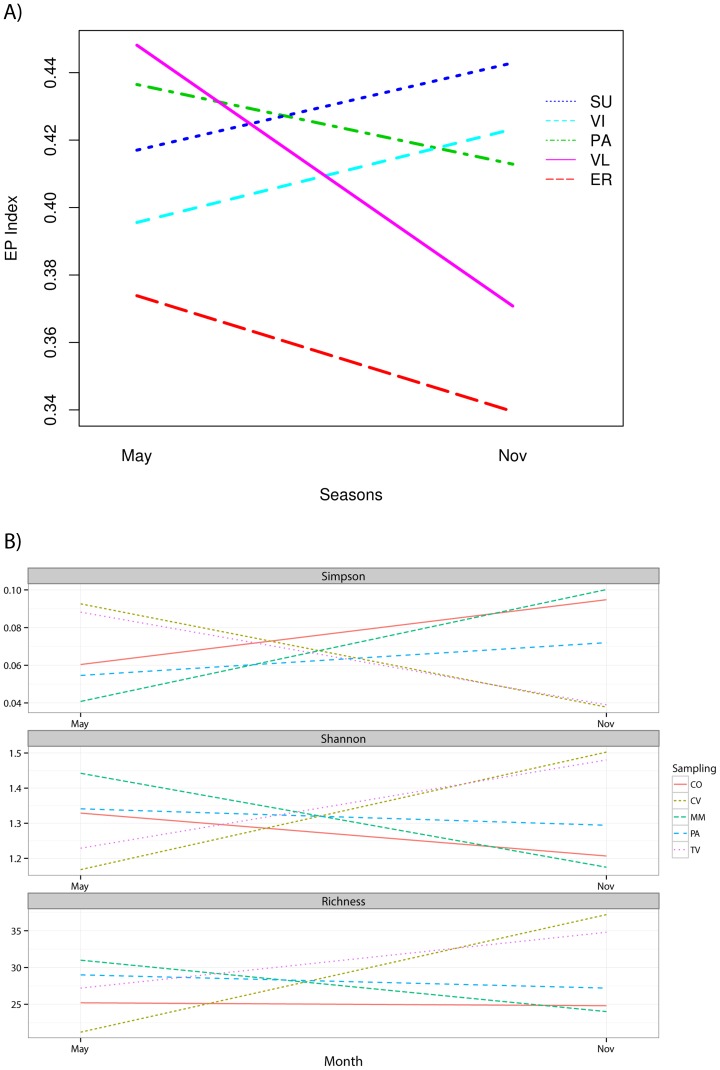
Effect of land-use and season on Eco-Physiological (EP) index of culturable bacteria (A) and diversity indices from CD-DGGE profiles (B).

The cultured bacteria belonging to fast-growing organisms, especially the r-strategists detected on day 2, dominated in all land uses and in both seasons (Table S4). The MM soil exhibited a lower EP index and a lower percentage of r-strategists in both seasons, possibly due to amensalism from a dominant bacterial group in the community. Variation of EP index of cultured bacteria was inspected using an ANOVA analysis of variance (Fig. 3A). Interestingly, statistical analysis revealed a significant effect of land uses on bacterial community structure (*P*<0.001) as well as an interaction effect between land uses and season change (*P*<0.001) on EP index. Variation of EP index due to seasonal changes was significant in the soil with higher human impact (TV) when compared with the other soils. Total bacterial concentrations varied significantly in respect of season (*P*<0.001) being higher in spring than in autumn (Table S2) in all but CO soils, with significant differences between CO and PA in spring, and between CO and MM, and MM and PA in autumn.

Overall results suggest that land uses affected cultured bacterial communities. Soils with low human impact (cork-oak forest) have a more stable bacterial density and show a less variation of bacterial community structure across seasons than soils subjected to high human impact such as tilled vineyard.

#### Bacterial community profiling by CD-DGGE and T-RFLP analyses

Culture dependent DGGE (CD-DGGE) fingerprinting of the 16S rRNA was used to characterize mixed bacterial communities recovered on agar plates. CD-DGGE represents a useful technique to follow the dynamics of distinct culturable fractions of the soil bacterial community in relation to physical, chemical and biological changes in the soil environment [60]. Since culture-dependent and culture-independent methods likely profile distinct fractions of the soil bacterial community with unique ecological roles [61], culturable bacteria may provide an ecologically relevant complement to culture-independent community characterization [62].

By pooling the bacterial cells growing on individual agar plates, we obtained a culture-dependent bacterial community. The analysis of DGGE profiles revealed clear banding patterns for each land-use and plant-cover types of sufficient complexity to investigate differences in soil microbial communities and identified stable communities with highly reproducible profiles (Fig. S2). A different community composition among land-use types was found as evidenced by the presence of different dominant signals; this finding indicated a compositional shift among soils examined in spring and autumn and subjected to different anthropogenic impact. Bacterial diversity was investigated through richness (*R*), Shannon-Weaver (*H’*), and Simpson (*D*) indices. Analysis of variance (ANOVA) confirmed the differences in the distribution of bacterial species due to land uses and sampling seasons (*P*<0.001). Both *R* and *H’* indices decreased from May to November in MM samples and increased in TV and CV samples, and the complementary opposite trend was observed for *D* index (Fig. 3B). Otherwise, cultured bacterial community in CO and PA soils did not vary significantly over seasons. Most likely, these results reflect the impact of both land-use and vegetation type and coverage on soil microbial communities. In particular, the low shift of biodiversity indices observed in CO and PA seems to be correlated to a higher stability of bacterial populations in natural habitats with low human impact, whereas populations inhabiting more anthropogenic areas tend to be more variable. Land-use type and, in particular, differences in vegetation dynamics may have a large role in modulating the temporal variability in soil bacterial communities. As observed by Lauber and co-workers [3], soils from the different land-use types did not exhibit identical temporal dynamics even though all the soils were located in close proximity and exposed to the same climatic conditions. Diversity indices obtained from T-RFLP analysis performed on total bacterial DNA (Fig. S3) partially confirmed the trends of diversity shown by CD-DGGE, though observed differences were not statistically significant (Table S5).

**Figure 4:**
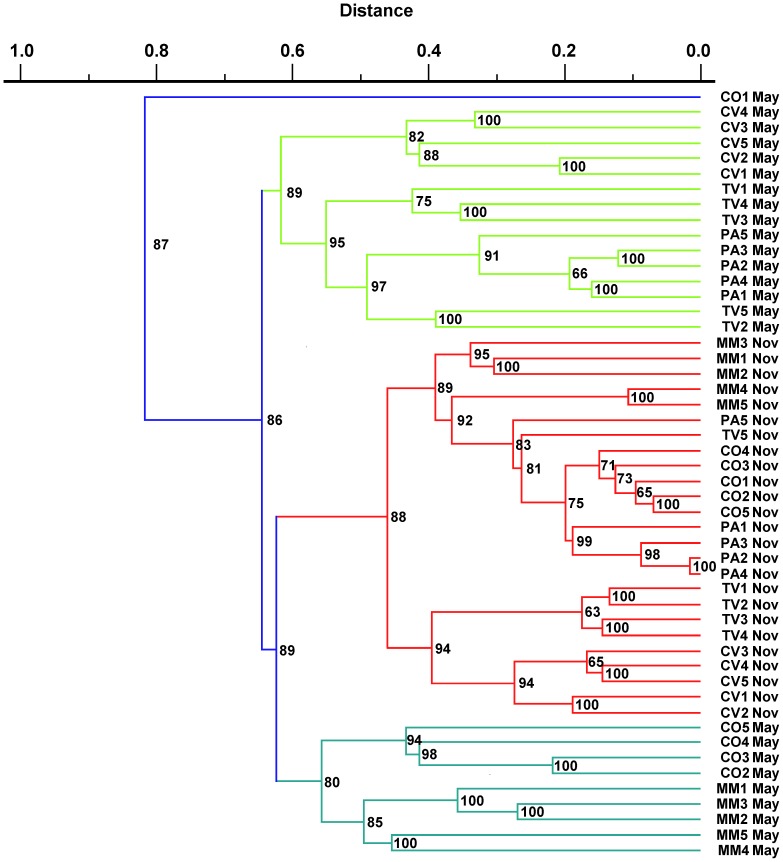
UPGMA dendrogram of DGGE profiles of amplified 16S rDNA of bacterial communities recovered in CO, PA, MM, CV and TV, in spring and autumn, generated using Phoretix ID advanced analysis package. Designation of samples is the same as in Fig. 1. The scale bar represents dissimilarity among samples. Consistency of each cluster was measured by the Cophenetic correlation coefficient shown at each node.

The unweighted pair-group method using arithmetic averages (UPGMA) of bacterial community profiling by CD-DGGE revealed a high diversity of the bacterial communities in each land-use and plant-cover soils. The similarity between the DGGE patterns of the soil bacterial communities revealed three distinct clusters (Fig. 4). Samples collected in autumn grouped into a separate cluster with about 52% similarity, whereas samples collected in spring grouped into two separate clusters, each of which composed by samples sharing about 40% similarity. The highest similarity values were shown by clusters based on soil land uses, suggesting a low bacterial variation within each soil type. This finding is consistent with previous reports showing that land-use type was the most important factor in determining the composition of soil microbial community [3].

The clustering results based on CD-DGGE were in agreement with culture-independent T-RFLP analysis that confirmed a different seasonal distribution of soil bacterial populations with subgroups associated to different land uses. Most of samples retrieved from the same soil (CO, PA, and MM) clustered together, whereas samples retrieved from TV and CV were often intermixed in the UPGMA dendrogram (Fig. S4). As culture-dependent and culture-independent profiles can separately resolve unique, diverse, and equally complex fractions of the soil bacterial community [60], our combined results from both CD-DGGE and T-RFLP methods, applied on culturable and total fractions, respectively, revealed an interaction effect between land uses and season change in affecting soil bacterial communities.

#### Taxa responses to land use determined by phylogenetic affiliation of soil bacterial isolates

Taxonomic affiliation was investigated on a total of 203 bacterial isolates. In detail, 20–21 colonies recovered from each soil sample at each season and showing different morphologies (for a total of 101 bacterial isolates in spring and 102 in autumn) were subjected to DNA extraction and PCR amplification of 16S rRNA gene. An amplicon of the expected size (about 1500 bp) was obtained from each isolate, and its nucleotide sequence was determined and submitted to GenBank (Table S6). Although it is generally accepted that not all bacteria, including types of soil bacteria, are culturable, the isolation of bacteria by agar plate cultivation and subsequent phylogenetic analysis permit to isolate and identify previously uncultured representatives or even new members of certain bacterial species for further analysis of their metabolic function. Therefore, even if the metagenome sequencing is becoming the most powerful tool to investigate microbial communities, the ability to isolate indigenous strains actually remains the unique way to further characterize and select soil bacteria showing interesting properties. Additionally, standard cultivation techniques have been shown to be able to capture members of the soil rare biosphere which could not be detected by metagenome sequencing [63].

The 203 sequences obtained were compared with those present in the GenBank databases by using the BLAST [64] program. Results showed that most of 16S rRNA gene sequences matched NCBI database sequences at 99–100% of similarity at the genus level, with *Arthrobacter*, *Bacillus*, *Stenotrophomonas*, *Pseudomonas* and *Burkholderia* as the most representative genera. So, identification at the genus level was achieved in 103 isolates and at the species level in 72 isolates, while 12 isolates were only affiliated to taxa level higher than genus and 16 remained unidentified (Table S6). Further comparison with GenBank databases by using the Seqmatch tool of the RDP indicated that 16S rRNA gene sequences were affiliated to four phyla: *Proteobacteria* (classes α, β and γ), *Bacteroidetes* (classes *Flavobacteria* and *Sphingobacteria*), *Actinobacteria* and *Firmicutes*. Even though the RDP analysis does not permit to affiliate a bacterial isolate to a given species, it allowed to assess the genus of almost all isolates, including those not identified through the BLAST search. In fact, a boostrap value of at least 80% is satisfactory for RDP requirement. Almost all our sequences gave rise to 100% boostrap, 12 ranged from 89% to 99%, and only two of them only resulted in low values (24 and 54% respectively, referred to two spring isolates).

**Figure 5:**
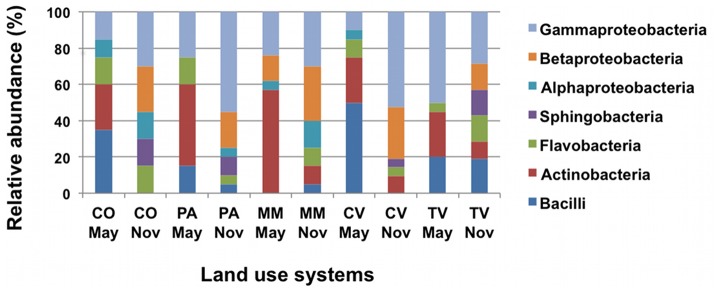
Relative abundances of major taxonomic groups across land use systems in spring and autumn. Detailed data of each class are listed in Additional file 1: Table S6. CO = cork-oak forest; PA = hayland-pasture rotation; MM = managed meadow; TV =  tilled vineyard; CV =  grass covered vineyard. Values presented are the mean percent.

**Figure 6:**
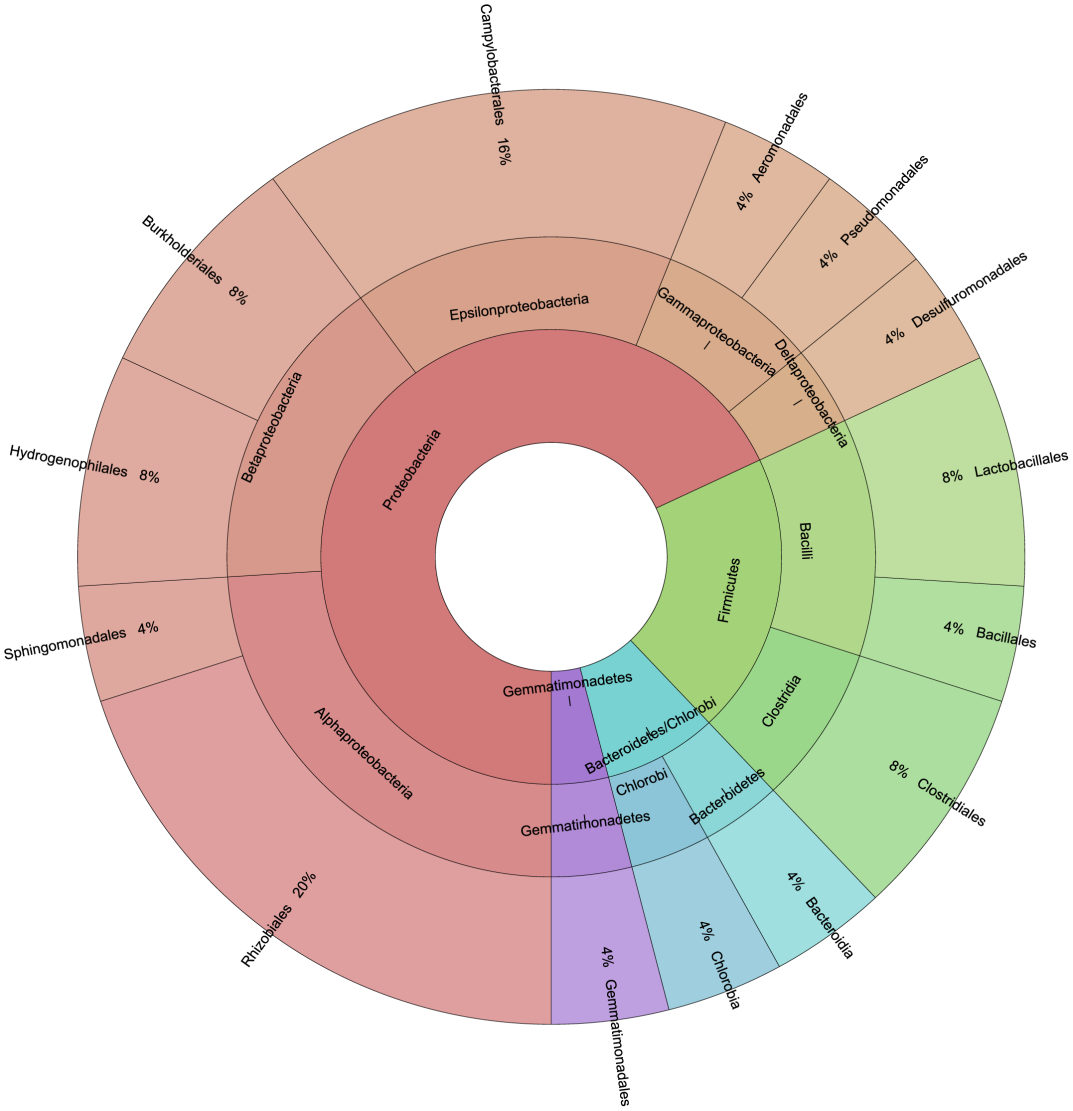
Plot of the taxonomic composition of total bacterial community as inferred from taxonomic interpretation of T-RFLP profiles.

Data obtained from both approaches allowed to assess that Berchidda soil is colonized by bacteria included in the classes of γ-*Proteobacteria* (with a prevalence of *Pseudomonas* and *Stenotrophomonas* genera), *Actinobacteria* (in particular, the genus *Arthrobacter*), β-*Proteobacteria* (with a prevalence of *Burkholderia* genus), *Bacilli* (with the genus *Bacillus* as the dominant one), Flavobacteria and Sphingobacteria. The relative abundances of the different classes identified by RDP across the different samples related to soil uses and season are represented in Figure 5. A putative taxonomic description of total bacterial community, derived from the interpretation of T-RFLP data (Fig. S3) is reported in Figure 6, in which the largest fraction of the community is represented by members of *Proteobacteria*, with α-*Proteobacteria* as the most abundant class.

Differences in class composition across the site were observed suggesting that the microbial composition changes in response to land uses. In fact, all the seven classes (α, β and γ *Proteobacteria*, *Sphingobacteria Flavobacteria*, *Actinobacteria* and *Bacilli*) were present in CV, PA, and CO soils, while all classes but α-*Proteobacteria* in TV and all classes but *Sphingobacteria* in MM were found. The observed large diffusion of *Proteobacteria* in all soil-uses is in agreement with previously reported data concerning soil bacterial communities in different land-use systems [65]. Within *Proteobacteria*, the majority of the isolates fell into the gamma subgroup, which showed higher relative abundance compared to that of any other taxa at the class level while a few were alfa proteobacteria, especially in vineyards and pasture. In all soil-uses, were also found *Actinobacteria*, in agreement with previous work [65], and β-*Proteobacteria*, like *Burkholderia* sp., previously detected by Pastorelli and co-workers [19] who analysed the denitrifying bacterial communities present in the same Berchidda soil samples. Among Bacteroidetes, it has to be noted the relative abundance of the genera *Chryseobacterium* sp. (in all soil uses) and *Flavobacterium* sp. (in all soil uses, but MM), which include isolates already observed in Korea soils [66], and bacteria with plant growth promoting properties recovered in Iran soil [67], respectively. As already pointed out by Fierer and co-workers [68], the β-*Proteobacteria* and *Bacteroidetes* follow copiotrophic lifestyles and their relative abundance were highest in soils with high C availability. In general, copiotrophic bacteria should have higher growth rates and traditional culturing methods are likely to select for microorganisms that can grow rapidly in high resource environments.

Strong differences in class composition were observed in each soil when sampled in the two seasons: for instance, *Bacilli* and *Actinobacteria* dominate in spring (particularly, *Bacilli* in CV and CO, while *Actinobacteria* in MM and PA), whilst β-*Proteobacteria* tend to dominate in autumn. Six out of the seven identified classes (β and γ-*Proteobacteria*, *Flavobacteria*, *Actinobacteria* and *Bacilli*) were recovered in both seasons whilst *Sphingobacteria* were recovered only in autumn. In all soils, *Actinobacteria* were prevalent in spring, β *Proteobacteria* predominated in autumn, while β and γ-*Proteobacteria* in both spring and autumn. When genera composition was used to infer the diversity indices of cultured bacteria (Table S7) an increase of diversity (estimated as Shannon H and Evenness) from May to November was present for all soils, but CO, where Shannon H was slightly higher in May than in November. In particular, MM soil showed the highest increases for most indices, especially for alpha diversity (*i.e.* the species richness and evenness within a sample) that has often been correlated with ecosystem stability and functionality [69]. It must be noted that cultivated bacterial populations did not fluctuate with seasonal changes in soils with low human input (cork-oak forest). The bacterial communities of the cork-oak forest soil were similar in richness and composition, furthering the point that in a community with moderate disturbance, new individuals and groups could be introduced in a manner that promotes competition and diversity of the community, thus establishing a more stable community [65].

These data suggest that shifts from forest to managed meadow and vineyard result in changes of bacterial communities composition. Previous studies have also shown that shifts from forest to grassland soil [70], from cultivated system to pasture [71] and from grazed pine forest to cultivated crop and grazed pasture [65] resulted in significant changes in bacterial community composition. Our results suggest that the use of culture-dependent 16S rRNA gene sequencing along with traditional analysis of soil physiochemical properties may provide insight into the ecological relevance of soil bacterial taxa.

## Conclusion

Overall, data obtained in this work revealed an important effect of land uses on both chemical and biochemical soil parameters. Soil bacterial communities were seasonally distinct and exhibited compositional shifts that tracked with changes in land use and soil management. This study, combining the pedological and biochemical data with microbiological and molecular analysis, furnishes a good methodological approach to describe the influence of different soil managements on soil microbial community structure. In fact, the results demonstrate that, in the same pedological conditions, long-term soil management influence the community structure; i.e., soils subjected to low human inputs (cork-oak forest and pasture) showed a more stable chemical and biochemical soil composition as well as bacterial community than those with high human input (vineyards and managed meadow). Further research is required to determine whether the observed shifts in bacterial community composition produce parallel changes in the functional attributes of these communities across soil types under different long-term management regimes. The use of culture-independent approaches, like metabarcoding and metagenome sequencing, will make it possible to identify the specific drivers of land-use dynamics exhibited by soil bacterial communities and to give a complete picture of the bacterial communities in a typical Mediterranean agro-silvo-pastoral system.

## Supporting Information

Figure S1 **Box-plot analysis showing the frequency distribution of physical-chemical and biological properties of the five Sardinia soils.**
(TIFF)

Figure S2 **Examples of CD-DGGE profiles of the soil bacterial communities associated to the different land uses in May and November.** A) From the left to right: hayland pasture rotation (PA), tilled vineyard (TV), grass covered vineyard (CV) in May; B) managed meadow (MM), cork-oak forest (CO), hayland pasture rotation (PA) in May; C) grass covered vineyard (CV) in May, grass covered vineyard (CV) in November, tilled vineyard (TV) in May; D) cork-oak forest (CO), hayland pasture rotation (PA), managed meadow (MM) in November.
(TIFF)

Figure S3 **Examples of T-RFLP profiles obtained after digestion with** ***Msp*** **I (A) and** ***Rsa*** **I (B) restriction enzymes of amplified of 16S rRNA gene sequences.**
(PDF)

Figure S4 **Cluster analysis of T-RFLP patterns generated by** ***Msp*** **I and** ***Rsa*** **I digestion of 16S rRNA gene sequences.** The UPGMA cluster analysis based on Jaccard similarity matrix was calculated for each set of samples using the “hclust” function of the R “stats” package. The scale bar represents the percent of dissimilarity.
(TIF)

Table S1 **Pedological profiles and classification of the soils investigated.**
(DOCX)

Table S2 **Determination of total organic carbon soil (C_org_), extractable carbon (C_ext_), humified carbon (C_HA+FA_), non humified carbon (C_NH_) and humification parameters of the five Sardinian soils.**
(DOCX)

Table S3 **Biochemical parameters measured in the five Sardinian soils.**
(DOCX)

Table S4 **r/k bacterial strategists, total culturable bacteria and EPI index measured in soils under different long-term management practices.**
(DOCX)

Table S5 **Diversity indices of total bacterial communities as inferred from T-RFLP profiles.**
(DOCX)

Table S6 **Phylogenetic affiliations of 203 randomly selected soil bacterial isolates based on comparative analysis of their 16S rRNA gene sequences.**
(DOCX)

Table S7 **The ratio of diversity indices related to the abundance of the different genera detected in cultured isolates between November and May.**
(DOCX)
