# Correction: Soil Bacterial Community Response to Differences in Agricultural Management along with Seasonal Changes in a Mediterranean Region

**DOI:** 10.1371/journal.pone.0124603

**Published:** 2015-04-10

**Authors:** The PLOS ONE Staff

There are errors in Fig 3, “Effect of land-use and season on Eco-Physiological (EP) index of culturable bacteria (A) and diversity indices from CD-DGGE profiles (B).” Please see the corrected Fig 3 here.

**Fig 3:**
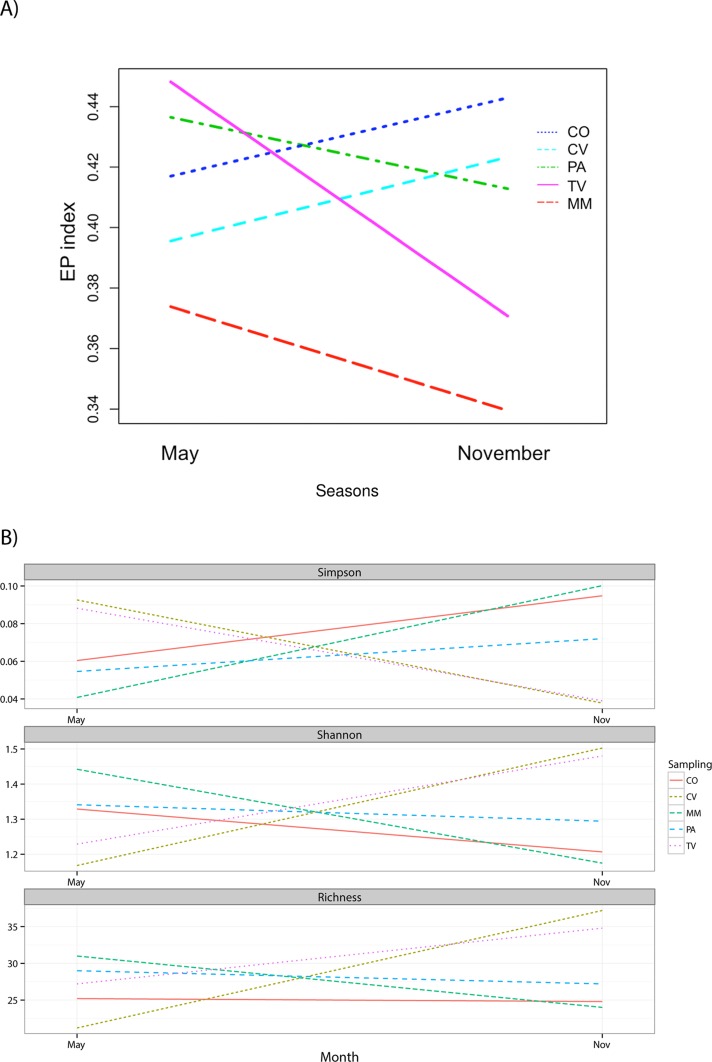
Effect of land-use and season on Eco-Physiological (EP) index of culturable bacteria (A) and diversity indices from CD-DGGE profiles (B).

## References

[1] Bevivino A, Paganin P, Bacci G, Florio A, Pellicer MS, Papaleo MC, et al (2014) Soil Bacterial Community Response to Differences in Agricultural Management along with Seasonal Changes in a Mediterranean Region. PLoS ONE 9(8): e105515 doi: 10.1371/journal.pone.0105515 25144665 10.1371/journal.pone.0105515 PMC4140800

