# Supplementary figures and images for: Soil Bacterial Community Response to Differences in Agricultural Management along with Seasonal Changes in a Mediterranean Region
Source: PLoS One. 2014 Aug 21;9(8):e105515. doi: 10.1371/journal.pone.0105515 (PMC4140800; doi:10.1371/journal.pone.0105515)

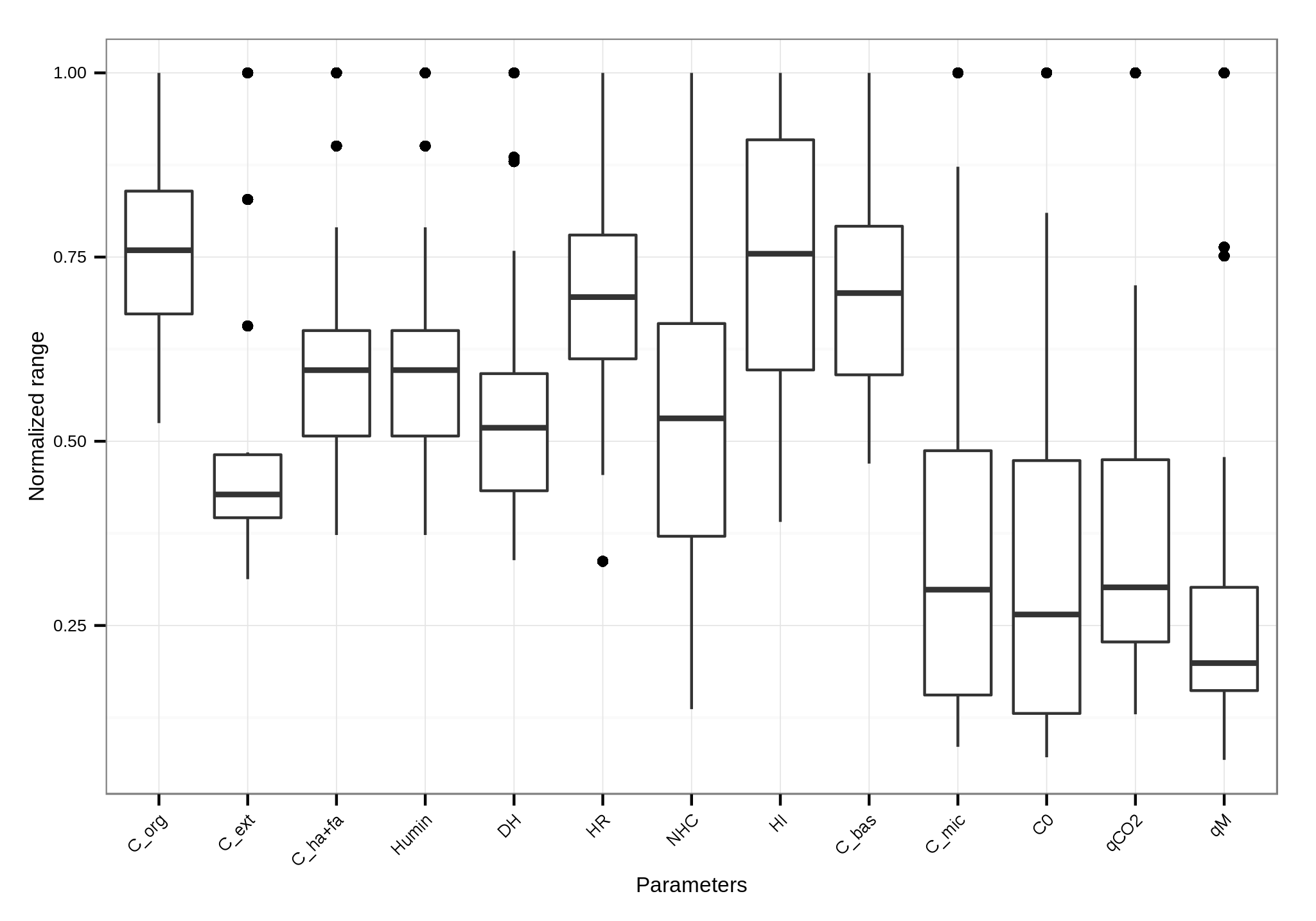

Supplement: Figure S1 — Box-plot analysis showing the frequency distribution of physical-chemical and biological properties of the five Sardinia soils. (TIFF) [file pone.0105515.s001.tiff]

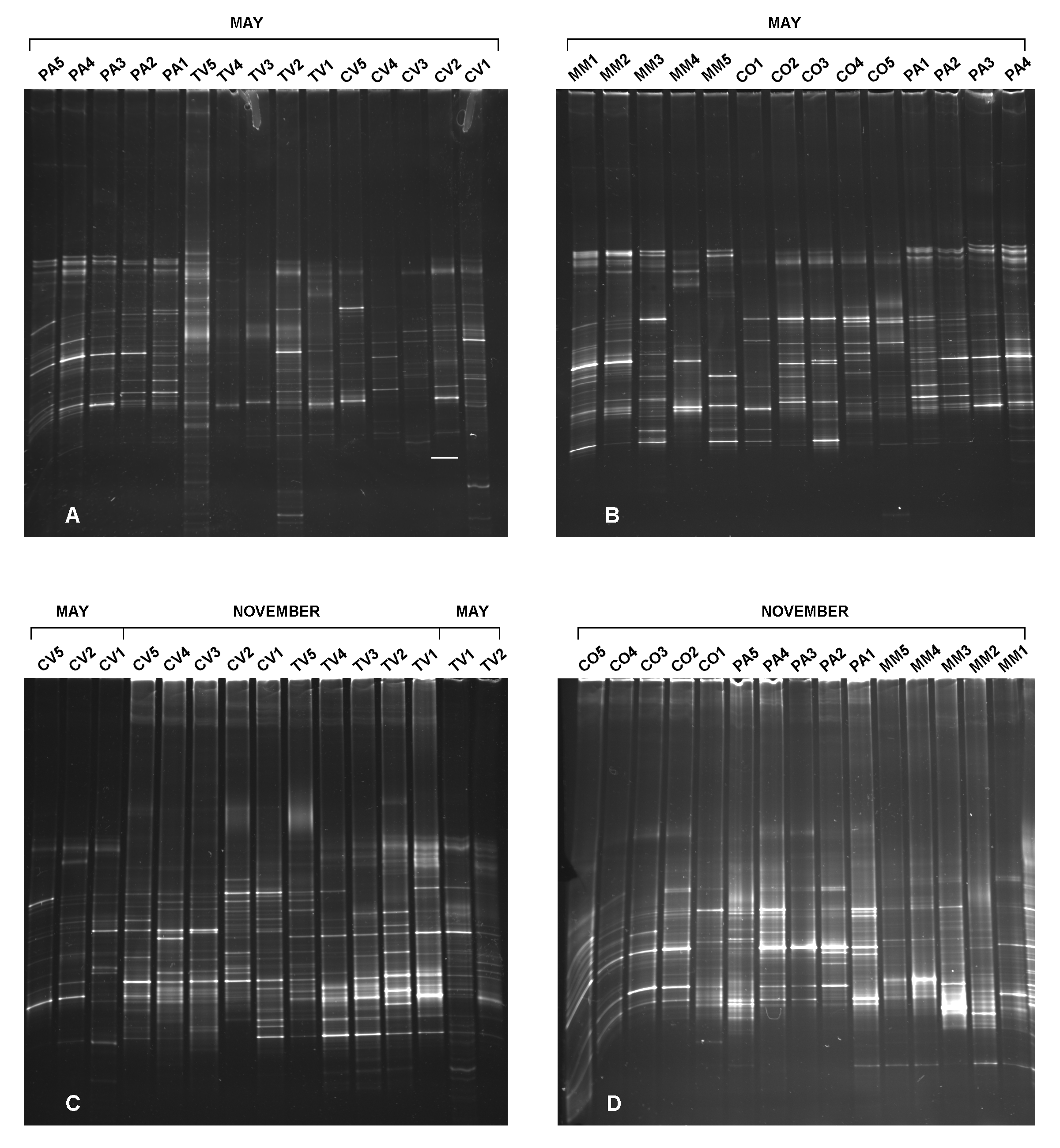

Supplement: Figure S2 — Examples of CD-DGGE profiles of the soil bacterial communities associated to the different land uses in May and November. A) From the left to right: hayland pasture rotation (PA), tilled vineyard (TV), grass covered vineyard (CV) in May; B) managed meadow (MM), cork-oak forest (CO), hayland pasture rotation (PA) in May; C) grass covered vineyard (CV) in May, grass covered vineyard (CV) in November, tilled vineyard (TV) in May; D) cork-oak forest (CO), hayland pasture rotation (PA), managed meadow (MM) in November. (TIFF) [file pone.0105515.s002.tiff]

A)

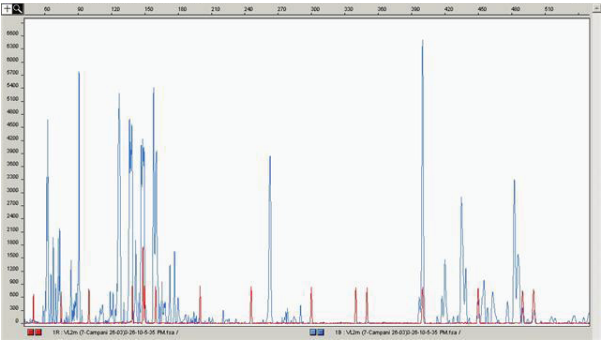

TV2

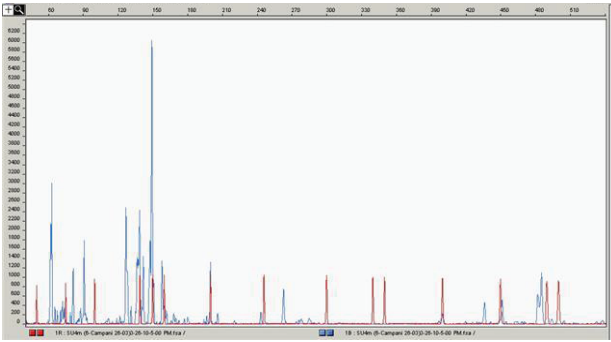

CO4

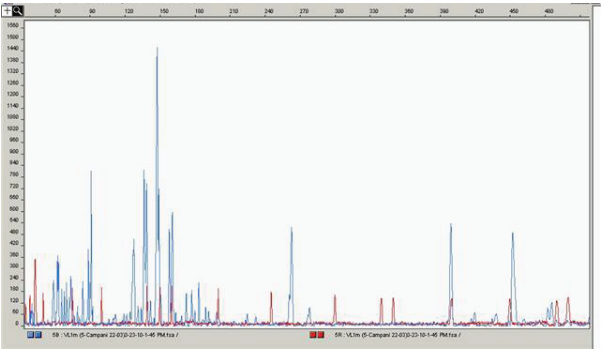

TV1

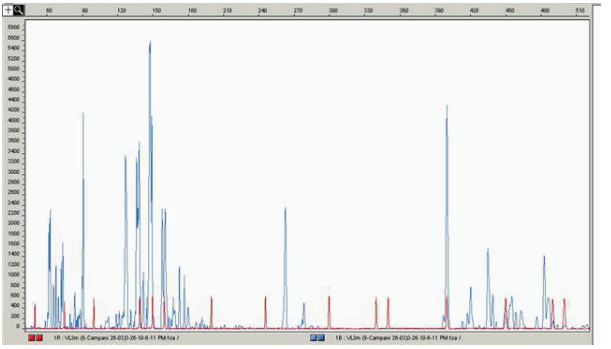

TV3

*MspI*

B)

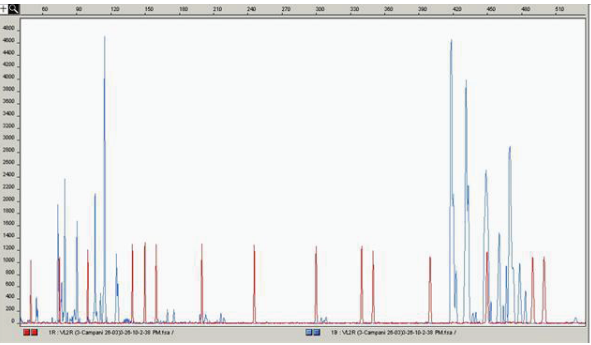

TV2

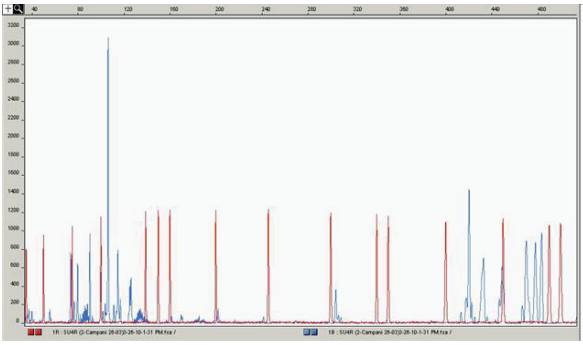

CO4

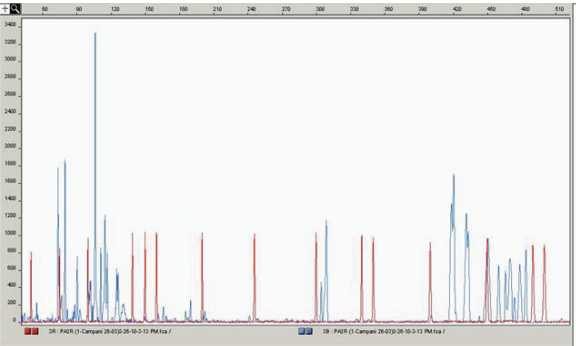

PA2

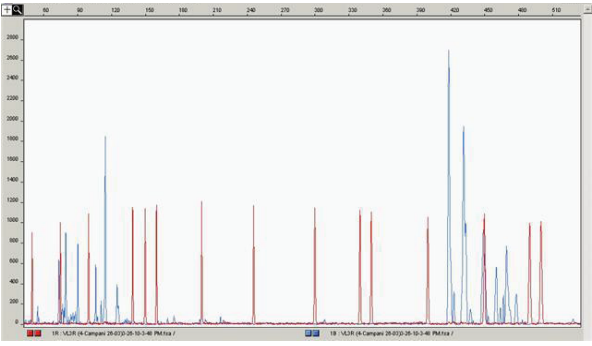

TV3

*RsaI*

Supplement: Figure S3 — Examples of T-RFLP profiles obtained after digestion with Msp I (A) and Rsa I (B) restriction enzymes of amplified of 16S rRNA gene sequences. (PDF) [file pone.0105515.s003.pdf]

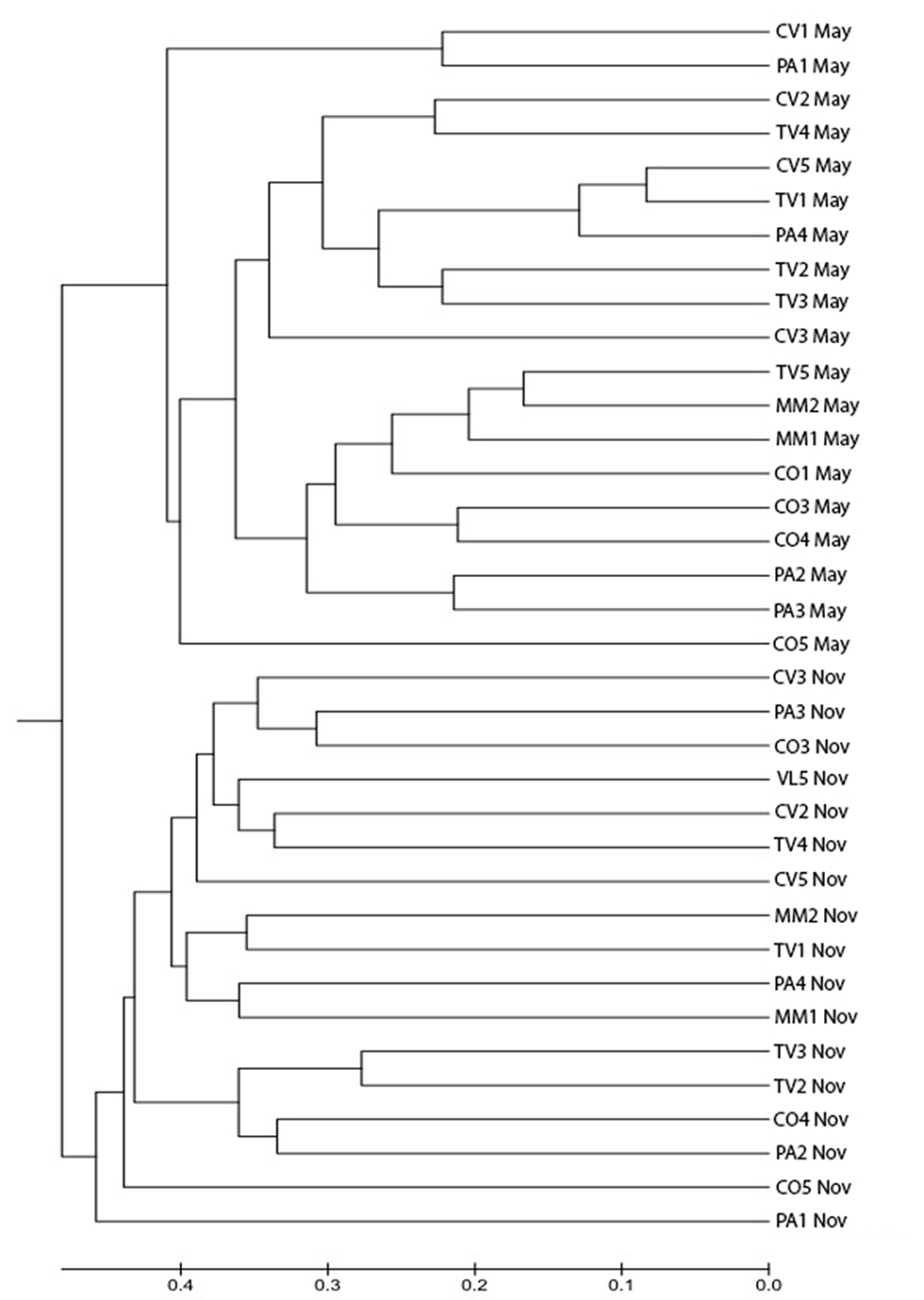

Supplement: Figure S4 — Cluster analysis of T-RFLP patterns generated by Msp I and Rsa I digestion of 16S rRNA gene sequences. The UPGMA cluster analysis based on Jaccard similarity matrix was calculated for each set of samples using the “hclust” function of the R “stats” package. The scale bar represents the percent of dissimilarity. (TIF) [file pone.0105515.s004.tif]
