# Supplementary material for: Soil Bacterial Community Response to Differences in Agricultural Management along with Seasonal Changes in a Mediterranean Region
Source: PLoS One. 2014 Aug 21;9(8):e105515. doi: 10.1371/journal.pone.0105515 (PMC4140800; doi:10.1371/journal.pone.0105515)
Supplement: Table S1 — Pedological profiles and classification of the soils investigated. (DOCX) [file pone.0105515.s005.docx]

Table S1. Pedological profiles and classification of the soils investigated.

| Soil uses^a^ | **Latitude N** | | **Longitude E** | **Average altitude** | **Slope** | **Exposition** | **Horizon** | | **Classification** |
| --- | --- | --- | --- | --- | --- | --- | --- | --- | --- |
|  |  | |  | **(m a.s.l.)** | **(%)** |  | **Type^b^** | **Thickness (cm)** | **USDA** |
| TV | 40° 49’ 22 | | 9° 18’ 04’’ | 282 | 1 – 4 | South - East | Ap | 0 - 20 |  |
|  |  | |  |  |  | (140° N) | Bw | 20 - 49 | Typic Dystroxerepts |
|  |  | |  |  |  |  | BC | 49 - 100 |  |
|  |  | |  |  |  |  | C | 100 - > 125 |  |
| CV | 40° 49’ 15’’ | | 9° 17’ 32’’ | 291 | 2 - 6 | South - East | Ap | 0 - 28 |  |
|  |  | |  |  |  | (120° N) | Bw | 28 - 78 | Typic Dystroxerepts |
|  |  | |  |  |  |  | C | 78 - 120 |  |
|  |  | |  |  |  |  | R | >120 |  |
| MM | 40° 48’ 58’’ | | 9° 17’ 20’’ | 297 | 6 - 18 | South - West | Ap | 0 - 46 |  |
|  |  | |  |  |  | (210° N) | Bw | 46 - 125 | Typic Dystroxerepts |
|  |  | |  |  |  |  | C | > 125 |  |
| PA | 40° 49’ 00’’ | | 9° 17’ 36’’ | 306 | 6 - 12 | North - East | Ap | 0 - 31 |  |
|  |  | |  |  |  | (40° N) | Bw | 31 - 77 | Typic Dystroxerepts |
|  |  | |  |  |  |  | C | 77 - 115 |  |
| CO | 40° 49’ 12’’ | | 9° 17’ 26’’ | 319 | 12 - 18 | South - East | A1 | 0 - 3 |  |
|  |  | |  |  |  | (130° N) | A2 | 3 - 15 | Lithic Xerorthents |
|  |  | |  |  |  |  | C1 | 15 - 40 |  |
|  | |  |  |  |  |  | C2 | 40 - 80 |  |

^a^Soil uses prefix [TV, tilled vineyard; CV, grass covered vineyard; MM, managed meadow; PA, hayland-pasture rotation; CO, cork-oak forest].

^b^Ap: surface soil, plowin; A1: distinctive layer within A surface soil; A2: distinctive layer within A surface soil; Bw: subsoil with distinctive color or structure without clay accumulation; BC: transition from subsoil to parent rock; C: parent rock; C1: distinctive layer within C parent rock; C2: distinctive layer within C parent rock; R: bedrock.
