# Supplementary material for: Soil Bacterial Community Response to Differences in Agricultural Management along with Seasonal Changes in a Mediterranean Region
Source: PLoS One. 2014 Aug 21;9(8):e105515. doi: 10.1371/journal.pone.0105515 (PMC4140800; doi:10.1371/journal.pone.0105515)
Supplement: Table S2 — Determination of total organic carbon soil (Corg), extractable carbon (Cext), humified carbon (CHA+FA), non humified carbon (CNH) and humification parameters of the five Sardinian soils. (DOCX) [file pone.0105515.s006.docx]

**Table S2. Determination of total organic carbon soil (C_org_), extractable carbon (C_ext_), humified carbon (C_HA+FA_), non humified carbon (C_NH_) and humification parameters of the five Sardinian soils.**

| **Land uses** | **C_org_ (%)** | **C_ext_ (%)** | **C_HA+FA_ (%)** | **C_NH_ (%)** | **Humin-C (%)** | **DH (%)** | **HR (%)** | **HI** |
| --- | --- | --- | --- | --- | --- | --- | --- | --- |
|  |  |  |  |  |  |  |  |  |
| **May** |  |  |  |  |  |  |  |  |
| **TV** | 1.65+0.21 | 1.07+0.23 | 0.65+0.13 | 0.42+0.13 | 0.58+0.11 | 61.42+6.22 | 39.31+2.59 | 0.64+0.17 |
| **CV** | 1.81+0.09 | 1.30+0.04 | 0.86+0.08 | 0.44+0.05 | 0.50+0.07 | 65.88+4.70 | 47.49+2.37 | 0.52+0.11 |
| **MM** | 1.41+0.27 | 1.24+0.22 | 0.66+0.23 | 0.57+0.04 | 0.18+0.05 | 52.49+10.28 | 45.95+8.23 | 0.96+0.42 |
| **PA** | 2.16+0.16 | 1.68+0.44 | 0.82+0.11 | 0.86+0.55 | 0.48+0.28 | 52.46+21.25 | 38.38+7.95 | 1.12+0.79 |
| **CO** | 2.51+0.53 | 2.04+0.43 | 0.94+0.25 | 1.10+0.21 | 0.47+0.09 | 46.02+3.41 | 37.32+3.09 | 1.18+0.16 |
|  |  |  |  |  |  |  |  |  |
| **November** |  |  |  |  |  |  |  |  |
| **TV** | 1.54+0.26 | 1.07+0.13 | 0.92+0.12 | 0.15+0.03 | 0.48+0.13 | 86.22+2.75 | 59.93+1.90 | 0.16+0.03 |
| **CV** | 1.53+0.18 | 0.98+0.09 | 0.80+0.07 | 0.18+0.02 | 0.55+0.09 | 81.87+0.63 | 52.58+1.38 | 0.22+0.01 |
| **MM** | 1.45+0.28 | 1.06+0.22 | 0.79+0.16 | 0.27+0.09 | 0.38+0.06 | 74.61+5.13 | 54.59+2.32 | 0.34+0.09 |
| **PA** | 2.01+0.20 | 1.53+0.31 | 0.90+0.05 | 0.63+0.30 | 0.48+0.32 | 60.59+12.85 | 44.88+0.55 | 0.70+0.76 |
| **CO** | 1.69+0.20 | 1.28+0.14 | 0.84+0.23 | 0.45+0.38 | 0.40+0.19 | 67.12+24.15 | 50.58+17.19 | 0.67+0.76 |

C_org_ = Total organic C

C_ext_ = Total extractable organic C

C_HA+FA_ = Humic-like acids fraction C + Fulvic-like acid fraction C

C_NH_: non-humified C

DH: degree of humification = [(C_HA+FA_)×100)/ C_ext_]

HR: humification rate = [(C_HA+FA_)×100)/ C_org_]

HI: humification index = C_NH_/(C_HA+FA_)
