# Supplementary material for: Soil Bacterial Community Response to Differences in Agricultural Management along with Seasonal Changes in a Mediterranean Region
Source: PLoS One. 2014 Aug 21;9(8):e105515. doi: 10.1371/journal.pone.0105515 (PMC4140800; doi:10.1371/journal.pone.0105515)
Supplement: Table S3 — Biochemical parameters measured in the five Sardinian soils. (DOCX) [file pone.0105515.s007.docx]

**Table S3. Biochemical parameters measured in the five Sardinian soils.**

| **Land uses** | **C_bas_** | **C_0_** | **C_mic_** | **qCO_2_** | **qM** |
| --- | --- | --- | --- | --- | --- |
|  | **(µg CO_2_-C g^-1^** | **(µg CO_2_-C g^-1^** | **(µg CO_2_-C g^-1^** | **(µg CO_2_-C_bas_** | **(µg CO_2_-C** |
|  | **dry soil)** | **dry soil)** | **dry soil)** | **µg^-1^ C_mic_ )h^-1^ 10^3^** | **µg^-1^ C)** |
|  |  |  |  |  |  |
| **May** |  |  |  |  |  |
| **TV** | 0.17+0.03 | 187.07+46.60 | 155.97+21.36 | 1.13+0.32 | 1.19+0.34 |
| **CV** | 0.21+0.02 | 264.81+22.13 | 244.34+74.25 | 0.92+0.27 | 1.47+0.16 |
| **MM** | 0.11+0.01 | 159.09+16.23 | 123.11+43.07 | 0.96+0.22 | 1.12+0.23 |
| **PA** | 0.13+0.01 | 157.20+35.33 | 151.81+26.42 | 0.84+0.17 | 0.74+0.19 |
| **CO** | 0.48+0.24 | 505.59+185.60 | 268.16+55.08 | 1.84+0.99 | 1.99+0.41 |
|  |  |  |  |  |  |
| **November** |  |  |  |  |  |
| **TV** | 0.13+0.03 | 149.47+15.57 | 52.04+7.20 | 2.48+0.83 | 1.05+0.19 |
| **CV** | 0.18+0.05 | 227.08+31.05 | 86.78+27.61 | 2.10+0.46 | 1.54+0.06 |
| **MM** | 0.06+0.01 | 104.26+11.26 | 34.35+5.18 | 1.91+0.43 | 0.79+0.21 |
| **PA** | 0.14+0.13 | 117.14+20.65 | 78.09+40.19 | 2.23+1.17 | 0.63+0.06 |
| **CO** | 0.49+0.02 | 494.99+186.74 | 119.37+69.74 | 4.72+1.57 | 2.73+0.71 |

C_bas_ = soil basal respiration

C_0_ = potentially mineralizable C

C_mic_ = soil microbial biomass C

*q*CO_2_ = metabolic quotient

*q*M = mineralization coefficient
