# Supplementary material for: Soil Bacterial Community Response to Differences in Agricultural Management along with Seasonal Changes in a Mediterranean Region
Source: PLoS One. 2014 Aug 21;9(8):e105515. doi: 10.1371/journal.pone.0105515 (PMC4140800; doi:10.1371/journal.pone.0105515)
Supplement: Table S4 — r/k bacterial strategists, total culturable bacteria and EPI index measured in soils under different long-term management practices. (DOCX) [file pone.0105515.s008.docx]

**Table S4. r/k bacterial strategists, total culturable bacteria and EPI index measured in soils under different long-term management practices.**

| Land uses | Culturable bacterial colony forming units recovered in 6 days^a,b^ | | | Total culturable | EPI index |
| --- | --- | --- | --- | --- | --- |
|  | **day 1** | **day 2** | **day 6** | **bacteria** |  |
| May |  |  |  |  |  |
| CO | 1.01x10^6^+7.87x10^5 aCF^ | 2.00x10^6^+1.13x10^6 aA^ | 1.49x10^6^+8.72x10^5 aBC^ | 4.50x10^6^+2.08x10^6^ | 0.42+0.02 |
| PA | 5.28x10^6^+2.99x10^6 abBEF^ | 1.02x10^7^+6.92x10^6 aA^ | 3.67x10^6^+9.53x10^5 bAC^ | 1.91x10^7^+1.01x10^7^ | 0.44+0.04 |
| MM | 1.50x10^6^+8.20x10^5 aCD^ | 6.43x10^6^+2.78x10^6 bA^ | 6.83x10^6^+6.89x10^6 abBC^ | 1.48x10^7^+7.92 x10^6^ | 0.37+0.06 |
| CV | 2.97x10^6^+8.09x10^5 aB^ | 2.24x10^6^+3.99x10^5 bA^ | 4.71x10^5^+4.90x10^4 cA^ | 5.68x10^6^+1.07x10^6^ | 0.40+0.02 |
| TV | 1.44x10^6^+5.12x10^5 aACDE^ | 3.29x10^6^+1.22x10^6 bA^ | 2.08x10^6^+3.00x10^5 cBC^ | 6.80x10^6^+1.88x10^6^ | 0.45+0.02 |
|  |  | | |  |  |
| November |  | | |  |  |
| CO | 1.23x10^6^+9.68x10^5 aBC^ | 1.27x10^6^+7.76x10^5 aA^ | 6.70x10^5^+6.38x10^5 bA^ | 3.18x10^6^+2.24x10^6^ | 0.44+0.02 |
| PA | 1.09x10^6^+7.74x10^5 aC^ | 1.39x10^6^ +1.28x10^6 aA^ | 4.12x10^5^+2.93x10^5 bA^ | 2.90x10^6^+1.97x10^6^ | 0.41+0.02 |
| MM | 2.24x10^4^+1.57x10^4 aA^ | 1.68x10^5^+6.57x10^4 bBC^ | 5.15x10^4^+2.11x10^4 cA^ | 2.42x10^5^+9.51x10^4^ | 0.34+0.01 |
| CV | 2.14x10^5^+1.85x10^5 aBC^ | 3.93x10^5^+2.36x10^5 bAC^ | 1.33x10^5^+4.21x10^4 aA^ | 7.40x10^5^+4.39x10^5^ | 0.42+0.03 |
| TV | 1.44x10^5^+1.12x10^5 aAB^ | 4.63x10^5^+1.91x10^5 bAC^ | 1.20x10^5^+1.25x10^4 aA^ | 7.26x10^5^+2.53x10^5^ | 0.37+0.04 |

^a^ Means + standard deviation. Means with the same letters are not significantly different (*P >*0*.*05), according to protected Fisher’s LSD test. Lowercase letters denote significant differences within each horizontal row, for each r/K class, and uppercase letters denote significant differences within each vertical column.

^b^ CFU recovered in day 1 and 2 are fast growers (r-like strategists) and those recovered on day 6 are slow growers (K-like strategists).

CO, cork-oak forest; PA, hayland-pasture rotation; MM, managed meadow; TV, tilled vineyard; CV, grass covered vineyard.
