# Supplementary material for: Soil Bacterial Community Response to Differences in Agricultural Management along with Seasonal Changes in a Mediterranean Region
Source: PLoS One. 2014 Aug 21;9(8):e105515. doi: 10.1371/journal.pone.0105515 (PMC4140800; doi:10.1371/journal.pone.0105515)
Supplement: Table S5 — Diversity indices of total bacterial communities as inferred from T-RFLP profiles. (DOCX) [file pone.0105515.s009.docx]

Table S5. Diversity indices of total bacterial communities as inferred from T-RFLP profiles.

| Diversity index | **CO** | | **PA** | | **MM** | | **CV** | | **TV** | |
| --- | --- | --- | --- | --- | --- | --- | --- | --- | --- | --- |
|  | **May** | **Nov** | **May** | **Nov** | **May** | **Nov** | **May** | **Nov** | **May** | **Nov** |
| Richness | 39.3±6.6 | 32.4±8.3 | 25.5±0.7 | 24.8±6.1 | 41.3±4.0 | 32.3±6.7 | 23.3±2.5 | 25.3±1.5 | 26.0±1.4 | 26±4.6 |
| Simpson (1-D) | 0.92±0.03 | 0.84±0.19 | 0.91±0.02 | 0.94±0.01 | 0.81±0.09 | 0.78±0.17 | 0.90±0.05 | 0.82±0.2 | 0.89±0.04 | 0.86±0.14 |
| Shannon (H’) | 3.31±0.28 | 3.28±0.17 | 3.10±0.21 | 3.02±0.24 | 3.05±0.16 | 2.56±0.69 | 3.02±0.12 | 2.60±0.69 | 3.13±0.17 | 2.68±0.45 |

Diversity indices related to the presence (Richness) and abundance (Shannon-Weaver diversity index H’ and Simpson Index of Diversity) of Terminal-Restriction Fragments in May and November are reported. Indices were computed with PAST software as previously reported [19]. CO, cork-oak forest; PA, hayland-pasture rotation; MM, managed meadow; CV, grass covered vineyard; TV, tilled vineyard.
