# Supplementary material for: Soil Bacterial Community Response to Differences in Agricultural Management along with Seasonal Changes in a Mediterranean Region
Source: PLoS One. 2014 Aug 21;9(8):e105515. doi: 10.1371/journal.pone.0105515 (PMC4140800; doi:10.1371/journal.pone.0105515)
Supplement: Table S6 — Phylogenetic affiliations of 203 randomly selected soil bacterial isolates based on comparative analysis of their 16S rRNA gene sequences. (DOCX) [file pone.0105515.s010.docx]

**Table S6.** **Phylogenetic affiliations of randomly selected soil bacterial isolates based on comparative analysis of their 16S rRNA gene sequences.**

| **Phylum^a^** | **Class^a^** | **Genus^a^** | **Isolate^b^** | **GenBank accession** | **Closest relative^c^** | **Accession number^c^** | **Identity^c^** |
| --- | --- | --- | --- | --- | --- | --- | --- |
| Firmicutes | Bacilli | Bacillus [100%] | CV1May | KJ482830 | Bacillus amyloliquefaciens HS8 | GU323369 | 99% |
|  |  | Bacillus [100%] | CV105May | KJ482845 | Bacillus amyloliquefaciens JS | HM055608 | 100% |
|  |  | Bacillus [100%] | TV53May | KJ482881 | Bacillus amyloliquefaciens DJFZ40 | GU568197 | 100% |
|  |  | Bacillus [100%] | CV13May | KJ482833 | Bacillus safensis Zw-22-2 | GU201861 | 100% |
|  |  | Bacillus [100%] | TV56May | KJ482889 | Bacillus safensis L10-1 | JF798363 | 100% |
|  |  | Bacillus [100%] | CV44May | KJ482842 | Bacillus megaterium BBST4 | HM119600 | 100% |
|  |  | Bacillus [100%] | CO5May | KJ482810 | Bacillus megaterium KU1 | JF683607 | 100% |
|  |  | Bacillus [100%] | CV37May | KJ482836 | Bacillus sp. I-116-14 | FJ786044 | 100% |
|  |  | Bacillus [100%] | CV49May | KJ482843 | Bacillus sp. HY 1.1 | FN692034 | 100% |
|  |  | Bacillus [100%] | CV50May | KJ482844 | Bacillus sp. BF47 | AM934688 | 99% |
|  |  | Bacillus [100%] | CV87bMay | KJ482847 | Bacillus sp. NCCP-158 | AB560659 | 100% |
|  |  | Bacillus [100%] | TV44Nov | KJ482902 | Bacillus sp. HY11(2010) | HM579802 | 99% |
|  |  | Bacillus [100%] | PA69May | KJ482765 | Bacillus sp. WN559 | DQ275174 | 99% |
|  |  | Bacillus [100%] | CO73May | KJ482825 | Bacillus sp. WN559 | DQ275174 | 99% |
|  |  | Bacillus [100%] | CO23May | KJ482814 | Bacillus sp. JG-TB10 | FR849922 | 99% |
|  |  | Bacillus [100%] | CV64May | KJ482838 | Bacillus sporothermodurans M215 | NR_026010 | 98% |
|  |  | Bacillus [100%] | TV82May | KJ482874 | Bacillus methylotrophicus Mo-Bm-14 | HQ662599 | 99% |
|  |  | Bacillus [100%] | PA41May | KJ482758 | Bacillus methylotrophicus BC79 | JF449444 | 100% |
|  |  | Bacillus [100%] | TV73aNov | KJ482897 | Bacillus anthracis CI | CP001746 | 99% |
|  |  | Bacillus [100%] | PA81May | KJ482767 | Bacillus cereus WA4-8 | JF496483 | 100% |
|  |  | Bacillus [100%] | CO16May | KJ482812 | Bacillus niacini G11 | FJ009401 | 99% |
|  |  | Bacillus [100%] | CO27May | KJ482816 | Bacillus simplex JH-3-4 | JF820101 | 100% |
|  |  | Bacillus [100%] | CO75May | KJ482826 | Bacillaceae bacterium SM17 | DQ195828 | 99% |
|  |  | Paenibacillus [100%] | TV53Nov | KJ482894 | Paenibacillus lautus MPF 112 | FN677987 | 99% |
|  |  | Paenibacillus [100%] | TV64Nov | KJ482910 | Paenibacillus sp. B17a | EU558286 | 99% |
|  |  | Paenibacillus [100%] | MM1Nov | KJ482730 | Uncult. bacterium TSCOR001_M13 | AB486660 | 96% |
|  |  | Paenibacillus [100%] | PA17Nov | KJ482772 | Paenibacillus sp. Gc62 | GU328679 | 99% |
|  |  | Brevibacillus [100%] | CV94May | KJ482849 | Brevibacillus brevis NBRC 100599 | AP008955 | 100% |
|  |  | Brevibacillus [100%] | CO44May | KJ482817 | Brevibacillus reuszeri DSM 9887T | AB112715 | 99% |
|  |  | Staphylococcus [100%] | TV73aMay | KJ482886 | Staphylococcus sp. Ap-9 | DQ644501 | 100% |
| Actinobacteria | Actinobacteria | Arthrobacter [100%] | CV4May | KJ482831 | Arthrobacter sp. P2 | DQ288888 | 99% |
|  |  | Arthrobacter [100%] | CV79May | KJ482839 | Arthrobacter sp. P2 | DQ288888 | 99% |
|  |  | Arthrobacter [100%] | CV11May | KJ482832 | Arthrobacter sp. B2 | AJ785759 | 100% |
|  |  | Arthrobacter [100%] | CV27bMay | KJ482834 | Arthrobacter sp. AGL 5 | EU118773 | 99% |
|  |  | Arthrobacter [100%] | MM4May | KJ482711 | Arthrobacter sp. SAZ1-3 | HQ236024 | 100% |
|  |  | Arthrobacter [100%] | MM12May | KJ482713 | Arthrobacter sp. S22242 | D84596 | 100% |
| **Phylum^a^** | **Class^a^** | **Genus^a^** | **Isolate^b^** | **GenBank accession** | **Closest relative^c^** | **Accession no.^c^** | **Identity^c^** |
| Actinobacteria | Actinobacteria | Arthrobacter [100%] | MM22May | KJ482715 | Arthrobacter sp. M3-4 | HQ425296 | 99% |
|  |  | Arthrobacter [100%] | MM40May | KJ482709 | Arthrobacter sp. KFC-73 | EF459536 | 99% |
|  |  | Arthrobacter [100%] | PA13May | KJ482753 | Arthrobacter sp. KFC-73 | EF459536 | 99% |
|  |  | Arthrobacter [100%] | PA30May | KJ482755 | Arthrobacter sp. KFC-73 | EF459536 | 99% |
|  |  | Arthrobacter [100%] | PA34May | KJ482756 | Arthrobacter sp. KFC-73 | EF459536 | 99% |
|  |  | Arthrobacter [100%] | PA72May | KJ482766 | Arthrobacter sp. Bt 06 | AJ971859 | 99% |
|  |  | Arthrobacter [100%] | MM51May | KJ482725 | Arthrobacter nicotinovorans SA36 | FN908773 | 100% |
|  |  | Arthrobacter [100%] | PA94May | KJ482769 | Arthrobacter nicotinovorans SA36 | FN908773 | 100% |
|  |  | Arthrobacter [100%] | PA63May | KJ482763 | Arthrobacter nicotinovorans TSWCSN20 | GQ284331 | 99% |
|  |  | Arthrobacter [100%] | MM80May | KJ482728 | Arthrobacter nitroguajacolicus WA1-7 | JF496467 | 100% |
|  |  | Arthrobacter [100%] | CO88May | KJ482827 | Arthrobacter nitroguajacolicus WA1-7 | JF496467 | 100% |
|  |  | Arthrobacter [100%] | PA8May | KJ482751 | Arthrobacter oryzae NBGD40 | HQ003445 | 100% |
|  |  | Arthrobacter [100%] | PA50May | KJ482760 | Arthrobacter gandavensis OS-44.c2 | AM237357 | 100% |
|  |  | Leucobacter [100%] | CV30May | KJ482835 | Leucobacter luti RF6T | AM072819 | 99% |
|  |  | Okibacterium [92%] | CV41Nov | KJ482854 | Microbacterium sp. VA8728_00 | AF306834 | 99% |
|  |  | Okibacterium [97%] | CV53Nov | KJ482856 | Microbacterium sp. VA8728_00 | AF306834 | 99% |
|  |  | Agromyces [100%] | TV11May | KJ482873 | Agromyces fucosus | AY158025 | 99% |
|  |  | Microbacterium [100%] | TV67May | KJ482888 | Microbacterium foliorum HR78 | JF700451 | 99% |
|  |  | Microbacterium [100%] | MM4Nov | KJ482731 | Microbact. hydrocarbonoxydans 1P10UE | EU977808 | 99% |
|  |  | Microbacterium [100%] | PA66May | KJ482764 | Microbacterium sp. JL1103 | DQ985063 | 99% |
|  |  | Microbacterium [100%] | CO46May | KJ482818 | Microbacterium oxydans IARI-M-13 | JF343232 | 100% |
|  |  | Microbacterium [100%] | CO56May | KJ482819 | Uncult.bacterium isolate BF0002A094 | AM696909 | 99% |
|  |  | Leifsonia [100%] | TV87May | KJ482890 | Actinobacterium kmd_217 | EU723155 | 99% |
|  |  | Leifsonia [100%] | TV99May | KJ482877 | Leifsonia xyli BZ1-1 | GU332619 | 98% |
|  |  | Leifsonia [100%] | TV13Nov | KJ482891 | Leifsonia sp. NaF-A-1 | FJ872398 | 99% |
|  |  | Leifsonia [100%] | CO21May | KJ482813 | Leifsonia poae VKM Ac-1401 | DQ232613 | 99% |
|  |  | Leifsonia [100%] | CO80May | KJ482823 | Leifsonia poae VKM Ac-1401 | DQ232613 | 100% |
|  |  | Plantibacter [100%] | TV103May | KJ482879 | Plantibacter sp. WPCB192 | FJ006927 | 99% |
|  |  | Plantibacter [100%] | MM82Nov | KJ482744 | Plantibacter sp. TSE8 | HM156125 | 99% |
|  |  | Cellulosimicrobium [100%] | TV30bNov | KJ482900 | Cellulosimicrobium cellulans CrK16 | GQ503328 | 99% |
|  |  | Williamsia [100%] | MM10May | KJ482712 | Williamsia sp. Tibet-IIU15 | DQ177476 | 99% |
|  |  | Williamsia [100%] | MM72May | KJ482726 | Williamsia sp. Tibet-IIU15 | DQ177476 | 99% |
|  |  | Curtobacterium [100%] | MM16May | KJ482714 | Curtobact. flaccumfaciens PDD-24b-7 | HQ256800 | 100% |
|  |  | Curtobacterium [100%] | MM37May | KJ482717 | Curtobact. flaccumfaciens PDD-24b-7 | HQ256800 | 100% |
|  |  | Curtobacterium [100%] | MM83May | KJ482720 | Arthrobacter nicotinovorans SA36 | FN908773 | 100% |
|  |  | Curtobacterium [100%] | MM90May | KJ482729 | Curtobacterium sp. WB20-13 | GU595335 | 100% |
| Bacteroidetes | Flavobacteria | Flavobacterium [100%] | CV41May | KJ482841 | Flavobacterium sp. PCK1 | GU078570 | 99% |
|  |  | Flavobacterium [100%] | TV3aMay | KJ482884 | Flavobacterium sp. R-32568 | AM403636 | 99% |
|  |  | Flavobacterium [100%] | PA37May | KJ482757 | Flavobacterium sp. WB3.2-27 | AM934658 | 99% |
| **Phylum^a^** | **Class^a^** | **Genus^a^** | **Isolate^b^** | **GenBank accession** | **Closest relative^c^** | **Accession no.^c^** | **Identity^c^** |
| Bacteroidetes | Flavobacteria | Flavobacterium [100%] | PA76Nov | KJ482786 | Flavobacterium sp. YO66 | DQ778318 | 98% |
|  |  | Flavobacterium [100%] | CO28Nov | KJ482795 | Flavobacterium sp. WB 4.3-19 | AM177629 | 99% |
|  |  | Chryseobacterium [100%] | CV108May | KJ482840 | Chryseobacterium joostei LMG 18208 | AY468479 | 98% |
|  |  | Chryseobacterium [100%] | TV41Nov | KJ482901 | Chryseobacterium sp. NX12 | EF601827 | 99% |
|  |  | Chryseobacterium 100%] | TV56aNov | KJ482895 | Chryseobacterium sp. NX12 | EF601827 | 99% |
|  |  | Chryseobacterium [100%] | TV93Nov | KJ482906 | Chryseobacterium sp. NX12 | EF601827 | 98% |
|  |  | Chryseobacterium [100%] | MM78Nov | KJ482743 | Chryseobacterium sp. NX12 | EF601827 | 98% |
|  |  | Chryseobacterium [100%] | MM85Nov | KJ482745 | Uncultured Bacteroidetes ATB-LH-5975 | FJ535138 | 99% |
|  |  | Chryseobacterium [100%] | PA10May | KJ482752 | Chryseobacterium sp. 3Ablue | EU057843 | 99% |
|  |  | Chryseobacterium [100%] | CO25May | KJ482815 | Chryseobacterium soli JS6-6 | EF591302 | 99% |
|  |  | Chryseobacterium [100%] | CO82May | KJ482824 | Chryseobacterium sp. CH33 | GU353129 | 99% |
|  |  | Chryseobacterium [100%] | CO68Nov | KJ482803 | Chryseobacterium sp. CH33 | GU353129 | 98% |
|  |  | Chryseobacterium [100%] | CO95May | KJ482828 | Chryseobacterium sp. COLI2 | EF442766 | 99% |
|  |  | Chryseobacterium [100%] | CO41Nov | KJ482798 | Chryseobacterium sp. PF-45 | FJ378901 | 98% |
|  |  | Chryseobacterium [71%] | CV30Nov | KJ482861 | Flavobacteriaceae bacterium TSBY-39 | DQ173003 | 99% |
|  |  | Epilithonimonas [54%] | PA84May | KJ482768 | Flavobacteriaceae bacterium TSBY-39 | DQ173003 | 98% |
|  | Sphingobacteria | Pedobacter [100%] | CV12Nov | KJ482864 | Pedobacter agri PB92 | EF660751 | 99% |
|  |  | Pedobacter [100%] | CO12Nov | KJ482793 | Pedobacter agri PB92 | EF660751 | 99% |
|  |  | Pedobacter [100%] | PA1Nov | KJ482776 | Pedobacter terrae QT16 | GU385862 | 99% |
|  |  | Pedobacter [100%] | PA33Nov | KJ482775 | Pedobacter sp. TB4-9-II | AY599663 | 97% |
|  |  | Pedobacter [100%] | CO36Nov | KJ482797 | Pedobacter sp. TPD41 | HM224489 | 99% |
|  |  | Pedobacter [100%] | CO84Nov | KJ482806 | Pedobacter cryoconitis B52 | EU169155 | 99% |
|  |  | Chitinophaga [100%] | TV4Nov | KJ482898 | Chitinophaga sp. HYLR39-2 | EU828457 | 99% |
|  |  | Sphingobacterium [100%] | TV87Nov | KJ482893 | Sphingobacterium multivorum DW-1 | EU240954 | 98% |
|  |  | Sphingobacterium [100%] | TV90Nov | KJ482905 | Sphingobacterium multivorum DW-1 | EU240954 | 98% |
| Proteobacteria | Gammaproteobacteria | Acinetobacter [100%] | CV75May | KJ482837 | Uncultured bacterium 16slp96-3a03.p1k | GQ158745 | 99% |
|  |  | Acinetobacter [100%] | CO100May | KJ482829 | Acinetobacter sp. NCCP 233 | AB619594 |  |
|  |  | Stenotrophomonas [100%] | CV85May | KJ482846 | Uncult.Stenotrophomonas sp. F1Sfeb.60 | GQ417086 | 99% |
|  |  | Stenotrophomonas [100%] | CV21Nov | KJ482851 | Stenotrophomonas sp. V | EU864327 | 99% |
|  |  | Stenotrophomonas [100%] | CV24Nov | KJ482852 | Stenotrophomonas sp. 1_2004 | AY563052 | 99% |
|  |  | Stenotrophomonas [96%] | CV67Nov | KJ482858 | Stenotrophomonas sp. 12C_21 | AY689084 | 99% |
|  |  | Stenotrophomonas [100%] | CV71Nov | KJ482865 | Stenotrophomonas chelatiphaga G-7 | FJ493060 | 99% |
|  |  | Stenotrophomonas [95%] | CV81Nov | KJ482859 | Stenotrophomonas sp. 1_2004 | AY563052 | 99% |
|  |  | Stenotrophomonas [97%] | TV49May | KJ482880 | Stenotrophomonas sp. 1_2004 | AY563052 | 99% |
|  |  | Stenotrophomonas [99%] | MM55Nov | KJ482739 | Stenotrophomonas sp. 1_2004 | AY563052 | 100% |
|  |  | Stenotrophomonas [100%] | MM25May | KJ482716 | Uncultured bacterium SedUMA28 | FJ849546 | 99% |
|  |  | Stenotrophomonas [100%] | MM10Nov | KJ482733 | Stenotrophomonas sp. 3C_5 | AY689032 | 99% |
|  |  | Stenotrophomonas [100%] | MM65May | KJ482722 | Stenotrophomonas sp. PDD-32b-72 | HQ256871 | 99% |
| **Phylum^a^** | **Class^a^** | **Genus^a^** | **Isolate^b^** | **GenBank accession** | **Closest relative^c^** | **Accession no.^c^** | **Identity^c^** |
| Proteobacteria | Gammaproteobacteria | Stenotrophomonas [100%] | PA44May | KJ482759 | Stenotrophomonas sp. PDD-32b-72 | HQ256871 | 99% |
|  |  | Stenotrophomonas [100%] | PA21Nov | KJ482773 | Stenotrophomonas sp. PDD-32b-72 | HQ256871 | 99% |
|  |  | Stenotrophomonas [100%] | CO10May | KJ482811 | Stenotrophomonas sp. PDD-32b-72 | HQ256871 | 100% |
|  |  | Stenotrophomonas [95%] | PA27Nov | KJ482774 | Stenotrophomonas sp. I_Gauze_K_8_5 | FJ267572 | 99% |
|  |  | Stenotrophomonas [100%] | PA44Nov | KJ482778 | Uncult. Stenotrophomonas sp. ASC45 | HQ912766 | 99% |
|  |  | Stenotrophomonas [100%] | PA53Nov | KJ482782 | Uncultured bacterium SedUMB1 | FJ849590 | 99% |
|  |  | Stenotrophomonas [97%] | PA59Nov | KJ482780 | Uncultured bacterium SedUMB1 | FJ849590 | 99% |
|  |  | Stenotrophomonas [98%] | PA67Nov | KJ482784 | Uncultured bacterium SedUMB1 | FJ849590 | 99% |
|  |  | Stenotrophomonas [100%] | PA81Nov | KJ482787 | Stenotrophomonas sp. 33T | HQ877451 | 99% |
|  |  | Enterobacter [100%] | CV87Nov | KJ482866 | Enterobacter sp. PR5 | GU086162 | 99% |
|  |  | Enterobacter [100%] | CV96bNov | KJ482860 | Enterobacter sp. CCBAU | DQ988938 | 99% |
|  |  | Enterobacter [100%] | TV47Nov | KJ482903 | Uncultured Pantoea sp. UBXB45 | GU569157 | 100% |
|  |  | Enterobacter [100%] | TV84Nov | KJ482904 | Enterobacter cloacae B9 | GQ421477 | 99% |
|  |  | Enterobacter [100%] | MM68Nov | KJ482748 | Enterobacter cloacae Bru-1 | HQ231214 | 99% |
|  |  | Enterobacter [100%] | PA84Nov | KJ482788 | Enterobacter cloacae Bru-1 | HQ231214 | 100% |
|  |  | Pseudomonas [100%] | CV4Nov | KJ482868 | Pseudomonas sp. K94.14 | AY456704 | 99% |
|  |  | Pseudomonas [100%] | CV7Nov | KJ482869 | Pseudomonas sp. 1/4_O_3 | EF540467 | 99% |
|  |  | Pseudomonas [100%] | CV27Nov | KJ482853 | Uncult.bacterium 16slp108-1g06.w2k | GQ157994 | 99% |
|  |  | Pseudomonas [100%] | TV25May | KJ482871 | Pseudomonadaceae bacterium zf-78-IV | AM292069 | 99% |
|  |  | Pseudomonas [100%] | TV37May | KJ482872 | Pseudomonadaceae bacterium zf-78-IV | AM292069 | 99% |
|  |  | Pseudomonas [100%] | TV61May | KJ482882 | Pseudomonadaceae bacterium zf-78-IV | AM292069 | 99% |
|  |  | Pseudomonas [100%] | TV21Nov | KJ482909 | Pseudomonadaceae bacterium zf-78-IV | AM292069 | 99% |
|  |  | Pseudomonas [100%] | TV84May | KJ482875 | Uncultured bacterium rRNA040 | AY958813 | 99% |
|  |  | Pseudomonas [100%] | TV34May | KJ482878 | Uncultured bacterium D23 | GQ389154 | 99% |
|  |  | Pseudomonas [100%] | TV96bMay | KJ482876 | Pseudomonas fluorescens LMG 5830 | GU198109 | 99% |
|  |  | Pseudomonas [100%] | TV107May | KJ482887 | Pseudomonas luteola Marseille | AY574976 | 99% |
|  |  | Pseudomonas [100%] | TV1Nov | KJ482892 | Pseudomonas putida isolate Tg | EU275363 | 99% |
|  |  | Pseudomonas [100%] | PA24Nov | KJ482777 | Pseudomonas putida ATCC 17494 | AF094740 | 100% |
|  |  | Pseudomonas [100%] | MM1May | KJ482710 | Pseudomonas sp. WR4-40 | FJ664279 | 100% |
|  |  | Pseudomonas [100%] | MM93May | KJ482723 | Pseudomonas sp. AD21 | DQ778036 | 99% |
|  |  | Pseudomonas [100%] | PA4May | KJ482750 | Pseudomonas sp. NZ011 | AY014803 | 100% |
|  |  | Pseudomonas [100%] | PA17May | KJ482754 | Pseudomonas sp. NCCP-18 | AB576763 | 99% |
|  |  | Pseudomonas [100%] | PA53May | KJ482761 | Pseudomonas sp. OS19 | EF491969 | 100% |
|  |  | Pseudomonas [100%] | PA56May | KJ482762 | Pseudomonas sp. BIM B-86 | GU784939 | 99% |
|  |  | Pseudomonas [100%] | PA70Nov | KJ482785 | Pseudomonas koreensis SSG5 | HM367599 | 98% |
|  |  | Pseudomonas [100%] | PA87Nov | KJ482789 | Pseudomonas sp. 3A_7 | AY689026 | 100% |
|  |  | Pseudomonas [100%] | CO32Nov | KJ482796 | Uncultured Pseudomonas sp. DC4 | EU169683 | 99% |
|  |  | Pseudomonas [100%] | CO81Nov | KJ482805 | Pseudomonas sp. 29H | EU057890 | 100% |
|  |  | Pseudomonas [100%] | CO92Nov | KJ482807 | Pseudomonas sp. 47A | GQ174494 | 99% |
| **Phylum^a^** | **Class^a^** | **Genus^a^** | **Isolate^b^** | **GenBank accession** | **Closest relative^c^** | **Accession no.^c^** | **Identity^c^** |
| Proteobacteria | Gammaproteobacteria | Pseudomonas [100%] | CO96Nov | KJ482808 | Pseudomonas lini B25 | EU169168 | 99% |
|  |  | Serratia [89%] | CV56Nov | KJ482857 | Uncultured bacterium FR_B_C2 | GQ443087 | 99% |
|  |  | Serratia [100%] | TV91May | KJ482883 | Serratia proteamaculans 568 | CP000826 | 99% |
|  |  | Serratia [100%] | TV24Nov | KJ482908 | Serratia proteamaculans | AJ233435 | 99% |
|  |  | Serratia [100%] | TV33Nov | KJ482911 | Serratia proteamaculans | AJ233435 | 99% |
|  |  | Serratia [100%] | CO60Nov | KJ482801 | Serratia proteamaculans wg-2 | EU627690 | 99% |
|  |  | Lysobacter [100%] | TV18May | KJ482885 | Lysobacter sp. ISE13 | EU034658 | 99% |
|  |  | Pantoea [94%] | MM59May | KJ482718 | Pantoea agglomerans BBPE277471 | FJ357811 | 99% |
|  |  | Pantoea [100%] | MM45Nov | KJ482737 | Pantoea agglomerans SR-1 | FJ593000 | 99% |
|  |  | Pantoea [100%] | MM66Nov | KJ482740 | Pantoea sp. II_Gauze_W_10_17 | FJ267564 | 99% |
|  |  | Raoultella [78%] | MM75Nov | KJ482742 | Klebsiella sp. HQ-3 | FJ432002 | 99% |
|  |  | Dyella [94%] | CO69May | KJ482822 | Uncultured Luteibacter sp. SMa210 | AM930508 | 99% |
|  |  | Erwinia [84%] | CO24Nov | KJ482794 | Erwinia sp. Y2(2010) | HM099662 | 99% |
|  | Alphaproteobacteria | Rhizobium [100%] | CV92May | KJ482848 | Agrobacterium tumefaciens AT108N | FJ666055 | 99% |
|  |  | Rhizobium [100%] | MM72Nov | KJ482741 | Rhizobium sp. PBSSR9 | JF327659 | 100% |
|  |  | Rhizobium [100%] | CO64Nov | KJ482802 | Rhizobium sp. PBSSR9 | JF327659 | 99% |
|  |  | Rhizobium [100%] | MM95Nov | KJ482747 | Rhizobium galegae SAFR-030 | AY167831 | 99% |
|  |  | Rhizobium [100%] | CO61May | KJ482821 | Agrobacterium rubi F266 | GU580894 | 99% |
|  |  | Rhizobium [100%] | CO4Nov | KJ482791 | Uncultured bacterium LC17 | FJ715980 | 99% |
|  |  | Mesorhizobium [100%] | MM28Nov | KJ482734 | Uncultured bacterium RB379 | AB240360 | 99% |
|  |  | Mesorhizobium [100%] | PA90bNov | KJ482779 | Uncultured bacterium : RB379 | AB240360 | 99% |
|  |  | Brevundimonas [100%] | MM68May | KJ482719 | Brevundimonas olei MJ15 | GQ250440 | 100% |
|  |  | Brevundimonas [100%] | CO1Nov | KJ482790 | Uncultured bacterium 9 | JF501072 | 99% |
|  |  | Bosea [100%] | CO60May | KJ482820 | Bosea sp. GSM-187 | FN600559 | 100% |
|  | Betaproteobacteria | Burkholderia [100%] | CV1Nov | KJ482850 | Burkholderia sp. CCBAU 25371 | HM107184 | 99% |
|  |  | Burkholderia [100%] | CV10Nov | KJ482867 | Burkholderia sp. CCBAU 25371 | HM107184 | 99% |
|  |  | Burkholderia [100%] | CV6Nov | KJ482863 | Burkholderia sp. Ellin138 | AF408980 | 99% |
|  |  | Burkholderia [100%] | CV13Nov | KJ482870 | Burkholderia sp. Ellin138 | AF408980 | 100% |
|  |  | Burkholderia [100%] | CV73Nov | KJ482862 | Burkholderia sp. Ellin138 | AF408980 | 100% |
|  |  | Burkholderia [100%] | TV27Nov | KJ482907 | Burkholderia sp. Ellin138 | AF408980 | 99% |
|  |  | Burkholderia [100%] | TV70Nov | KJ482896 | Burkholderia sp. Ellin138 | AF408980 | 99% |
|  |  | Burkholderia [100%] | MM22Nov | KJ482735 | Burkholderia sp. Ellin138 | AF408980 | 99% |
|  |  | Burkholderia [100%] | MM32Nov | KJ482736 | Burkholderia caryophylli ATCC 25418T | AB021423 | 98% |
|  |  | Burkholderia [100%] | PA10Nov | KJ482770 | Burkholderia sp. Y86 | FJ772045 | 99% |
|  |  | Burkholderia [99%] | PA13Nov | KJ482771 | Burkholderia glathei N15 | NR_037065 | 97% |
|  |  | Burkholderia [90%] | PA61Nov | KJ482783 | Burkholderia sp. YEX124 | AB558176 | 97% |
|  |  | Burkholderia [100%] | CO52Nov | KJ482799 | Burkholderia phytofirmans PW136 | JF494821 | 98% |
|  |  | Burkholderia [100%] | CO56Nov | KJ482800 | Burkholderia phytofirmans isolate PSB48 | HQ242761 | 98% |
|  |  | Burkholderia [100%] | CO72Nov | KJ482804 | Uncultured Burkholderia sp. 83 | JF500960 | 100% |
| **Phylum^a^** | **Class^a^** | **Genus^a^** | **Isolate^b^** | **GenBank accession** | **Closest relative^c^** | **Accession no.^c^** | **Identity^c^** |
| Proteobacteria | Betaproteobacteria | Burkholderia [100%] | CO100Nov | KJ482809 | Burkholderia sp. D22(2010) | HM624029 | 99% |
|  |  | Variovorax [100%] | CV50Nov | KJ482855 | Uncultured bacterium SedUMA5 | FJ849570 | 99% |
|  |  | Variovorax [100%] | MM43Nov | KJ482749 | Variovorax sp. CYEB-15 | FJ422402 | 100% |
|  |  | Variovorax [100%] | CO8Nov | KJ482792 | Uncultured Variovorax sp. T301B6 | HM438647 | 99% |
|  |  | Shinella [100%] | TV7Nov | KJ482899 | Uncultured bacterium aab68d11 | DQ814424 | 99% |
|  |  | Pusillimonas [24%] | MM77May | KJ482727 | Uncultured bacterium FS343 | FN667020 | 99% |
|  |  | Delftia [100%] | MM97May | KJ482724 | Bacterium A134(2011) | HQ849070 | 100% |
|  |  | Delftia [100%] | MM100May | KJ482721 | Bacterium A134(2011) | HQ849070 | 100% |
|  |  | Achromobacter [100%] | MM7Nov | KJ482732 | Achromobacter spanius CCM7183T | FM999732 | 99% |
|  |  | Achromobacter [100%] | MM93Nov | KJ482746 | Achromobacter spanius, CCM7183T | FM999732 | 100% |
|  |  | Achromobacter [100%] | PA50Nov | KJ482781 | Uncultured Achromobacter sp. F7aug.25 | GQ416598 | 99% |
|  |  | Tetrathiobacter [100%] | MM53Nov | KJ482738 | Uncultured Alcaligenes sp. 16 | FJ195782 | 99% |

^a^Identification performed with RDP Classification Algorithm. Bootstrap confidence values are 100% for Phylum and Class and are given between brackets for Genus (classification is well supported for confidence > 80%).

^b^Soil type prefix [CO, cork-oak forest; PA, hayland-pasture rotation; MM, managed meadow; TV, tilled vineyard; CV, grass covered vineyard] followed by the number and month of sampling.

^c^Sequence similarities between 16SrDNA gene sequences of isolates and those of the closest relatives in the NCBI database.
