# Supplementary material for: Soil Bacterial Community Response to Differences in Agricultural Management along with Seasonal Changes in a Mediterranean Region
Source: PLoS One. 2014 Aug 21;9(8):e105515. doi: 10.1371/journal.pone.0105515 (PMC4140800; doi:10.1371/journal.pone.0105515)
Supplement: Table S7 — The ratio of diversity indices related to the abundance of the different genera detected in cultured isolates between November and May. (DOCX) [file pone.0105515.s011.docx]

Table S7. The ratio of diversity indices related to the abundance of the different genera detected in cultured isolates between November and May.

| Diversity index | **CO** | **PA** | **MM** | **CV** | **TV** |
| --- | --- | --- | --- | --- | --- |
| Taxa richness | 0.91 | 1.00 | 1.56 | 1.13 | 1.09 |
| Simpson 1-D | 1.02 | 1.13 | 1.11 | 1.06 | 1.10 |
| Shannon H | 0.99 | 1.15 | 1.31 | 1.11 | 1.17 |
| Eveness e^H/S | 1.08 | 1.30 | 1.18 | 1.08 | 1.29 |
| Fisher Alpha | 0.79 | 0.95 | 3.47 | 1.27 | 1.16 |
| Chao-1 | 0.74 | 0.37 | 1.88 | 0.78 | 0.51 |

Indices were computed with PAST software [42]. In green, values <1; in red values >1; in orange, values=1. CO, cork-oak forest; PA, hayland-pasture rotation; MM, managed meadow; TV, tilled vineyard; CV, grass covered vineyard.
